# Comparative Genomics Provides Insights into the Taxonomy of *Azoarcus* and Reveals Separate Origins of *Nif* Genes in the Proposed *Azoarcus* and *Aromatoleum* Genera

**DOI:** 10.3390/genes12010071

**Published:** 2021-01-07

**Authors:** Roberto Tadeu Raittz, Camilla Reginatto De Pierri, Marta Maluk, Marcelo Bueno Batista, Manuel Carmona, Madan Junghare, Helisson Faoro, Leonardo M. Cruz, Federico Battistoni, Emanuel de Souza, Fábio de Oliveira Pedrosa, Wen-Ming Chen, Philip S. Poole, Ray A. Dixon, Euan K. James

**Affiliations:** 1 Laboratory of Artificial Intelligence Applied to Bioinformatics, Professional and Technical Education Sector—SEPT, UFPR, Curitiba, PR 81520-260, Brazil; 2 Department of Biochemistry and Molecular Biology, UFPR, Curitiba, PR 81531-980, Brazil; camillareginatto.p@gmail.com (C.R.D.P.); leonardo@ufpr.br (L.M.C.); souzaem@ufpr.br (E.d.S.); fpedrosa@ufpr.br (F.d.O.P.); 3 The James Hutton Institute, Invergowrie, Dundee DD2 5DA, UK; Marta.Maluk@hutton.ac.uk; 4 John Innes Centre, Department of Molecular Microbiology, Norwich NR4 7UH, UK; Marcelo.Batista@jic.ac.uk; 5 Centro de Investigaciones Biológicas Margarita Salas-CSIC, Department of Biotechnology of Microbes and Plants, Ramiro de Maeztu 9, 28040 Madrid, Spain; mcarmona@cib.csic.es; 6 Faculty of Chemistry, Biotechnology and Food Science, NMBU—Norwegian University of Life Sciences, 1430 Ås, Norway; madanbiotek@gmail.com; 7 Laboratory for Science and Technology Applied in Health, Carlos Chagas Institute, Fiocruz, Curitiba, PR 81310-020, Brazil; helisson.faoro@fiocruz.br; 8 Department of Microbial Biochemistry and Genomics, IIBCE, Montevideo 11600, Uruguay; fbattistoni@iibce.edu.uy; 9 Laboratory of Microbiology, Department of Seafood Science, NKMU, Kaohsiung City 811, Taiwan; p62365@ms28.hinet.net; 10 Department of Plant Sciences, University of Oxford, South Parks Road, Oxford OX1 3RB, UK; philip.poole@plants.ox.ac.uk

**Keywords:** *Azoarcus*, *Aromatoleum*, nitrogen fixation, *nif* genes, *Thauera*, plant colonisation

## Abstract

Among other attributes, the Betaproteobacterial genus *Azoarcus* has biotechnological importance for plant growth-promotion and remediation of petroleum waste-polluted water and soils. It comprises at least two phylogenetically distinct groups. The “plant-associated” group includes strains that are isolated from the rhizosphere or root interior of the C4 plant Kallar Grass, but also strains from soil and/or water; all are considered to be obligate aerobes and all are diazotrophic. The other group (now partly incorporated into the new genus *Aromatoleum*) comprises a diverse range of species and strains that live in water or soil that is contaminated with petroleum and/or aromatic compounds; all are facultative or obligate anaerobes. Some are diazotrophs. A comparative genome analysis of 32 genomes from 30 *Azoarcus-Aromatoleum* strains was performed in order to delineate generic boundaries more precisely than the single gene, 16S rRNA, that has been commonly used in bacterial taxonomy. The origin of diazotrophy in *Azoarcus-Aromatoleum* was also investigated by comparing full-length sequences of *nif* genes, and by physiological measurements of nitrogenase activity using the acetylene reduction assay. Based on average nucleotide identity (ANI) and whole genome analyses, three major groups could be discerned: (i) *Azoarcus* comprising *Az. communis*, *Az. indigens* and *Az. olearius*, and two unnamed species complexes, (ii) *Aromatoleum* Group 1 comprising *Ar. anaerobium*, *Ar. aromaticum*, *Ar. bremense*, and *Ar. buckelii*, and (iii) *Aromatoleum* Group 2 comprising *Ar. diolicum*, *Ar. evansii*, *Ar. petrolei*, *Ar. toluclasticum*, *Ar. tolulyticum*, *Ar. toluolicum,* and *Ar. toluvorans*. Single strain lineages such as *Azoarcus* sp. KH32C, *Az. pumilus,* and *Az. taiwanensis* were also revealed. Full length sequences of *nif*-cluster genes revealed two groups of diazotrophs in *Azoarcus-Aromatoleum* with *nif* being derived from *Dechloromonas* in *Azoarcus sensu stricto* (and two *Thauera* strains) and from *Azospira* in *Aromatoleum* Group 2. Diazotrophy was confirmed in several strains, and for the first time in *Az. communis* LMG5514, *Azoarcus* sp. TTM-91 and *Ar. toluolicum* T^T^. In terms of ecology, with the exception of a few plant-associated strains in *Azoarcus* (s.s.), across the group, most strains/species are found in soil and water (often contaminated with petroleum or related aromatic compounds), sewage sludge, and seawater. The possession of *nar*, *nap*, *nir*, *nor,* and *nos* genes by most *Azoarcus-Aromatoleum* strains suggests that they have the potential to derive energy through anaerobic nitrate respiration, so this ability cannot be usefully used as a phenotypic marker to distinguish genera. However, the possession of *bzd* genes indicating the ability to degrade benzoate anaerobically plus the type of diazotrophy (aerobic vs. anaerobic) could, after confirmation of their functionality, be considered as distinguishing phenotypes in any new generic delineations. The taxonomy of the *Azoarcus-Aromatoleum* group should be revisited; retaining the generic name *Azoarcus* for its entirety, or creating additional genera are both possible outcomes.

## 1. Introduction

The genus *Azoarcus (Az.)* was first discovered after several strains were isolated from surface sterilised roots of the halo-tolerant C4 Poaceae species *Leptochloa fusca* L. (Kallar Grass) in Pakistan [1,2]. The new species *Az. communis* and *Az. indigens* were subsequently described [3], and the model endophytic *Azoarcus* strain, BH72, which was also isolated from Pakistani Kallar Grass, was recently incorporated into the new species, *Az. olearius* [4] on the basis of its high genomic similarity to the type strain, DQS-4^T^ (which was isolated from oil-contaminated soil; [5]). Subsequent to the discovery of the plant-associated *Azoarcus* spp., several new species were isolated from non-plant sources, particularly from soil and/or water contaminated with aromatic compounds such as *Az. evansii*, *Az. taiwanensis*, *Az. tolulyticus*, *Az. toluvorans*, *Az. toluclasticus*, and *Az. buckelii* [6,7,8,9,10,11] as well as several strains that have not yet been classified/described to species level [12,13,14,15,16]. These are taxonomically separate from the plant-associated species [4,5,12,14,17,18] due to their ability to metabolize aromatic compounds, many of them under strict anaerobic conditions, and hence may have biotechnological potential as agents for the remediation of oil-polluted soils [19,20]. In addition, unlike the plant-associated species that are considered to be obligate aerobes, they are facultative anaerobes that can use nitrate as an alternative electron acceptor under anoxic conditions [21]. Both *Azoarcus* groups contain diazotrophs, but diazotrophy is not as common in the non-plant-associated group, and the *nif* genes of the two groups are unrelated [5,14,20,22].

So different are the two groups in terms of their molecular phylogeny and their lifestyles that there have been proposals to separate them into two genera [5,12,17,20]. Accordingly, Rabus et al. [21] have recently re-organised *Azoarcus* and described a new genus, *Aromatoleum*, comprising all species outside the “plant-associated” group including several newly-described species (*Ar. aromaticum*, *Ar. bremense*, *Ar. diolicum*, *Ar. petrolei*, and *Ar. toluolicum*). *Azoarcus* now consists only of the species *Az. communis*, *Az. indigens,* and *Az. olearius*. However, the molecular taxonomy in the Rabus et al. [21] study was based solely on 16S rRNA sequences and did not include several *Azoarcus* strains that were either not currently allocated to a species, or have been done so invalidly (e.g., *Az. taiwanensis*). Shortly after publication of Rabus et al. [21], one of these strains, SY39, which was isolated from seawater, was described as the type strain of a new species, *Az. pumilus*, that clusters loosely with *Az. taiwanensis*, and which is intermediate between *Azoarcus* and *Aromatoleum* [23], thus increasing uncertainty about strict taxonomic divisions in *Azoarcus sensu lato*. Rapid technical advances combined with modern computational solutions have greatly improved the speed and quality of the analysis and sequencing of bacterial genomes, concomitantly with a huge reduction in costs. Such genome sequences can be used to more accurately discern the phylogeny/taxonomy of groups of bacteria as the full sequences of several core genes can be used to construct robust phylogenies. Recent examples are *Burkholderia-Paraburkholderia-Caballeronia* [24], *Trinickia* and *Mycetohabitans* [25], and *Bradyrhizobium* [26,27].

The aim of the present study was to construct a robust phylogeny, utilising whole genome sequences of all described *Azoarcus* and *Aromatoleum* species together with genomes of strains that have not yet been allocated species status. A further aim was to use the genomes to investigate the lifestyle and ecology of the strains, with particular emphasis on the origin of the different types of *nif* genes in *Azoarcus-Aromatoleum*.

## 2. Materials and Methods

### 2.1. Bacterial Strains and Genomes

Fourteen *Azoarcus-Aromatoleum* genomes were sequenced as part of this study (Table S1a,b). These, together with previously published genome sequences and with others available in the National Center for Biotechnology Information (NCBI) database, totalling 32 *Azoarcus-Aromatoleum* genomes (Table 1) allowed robust reanalysis of the *Azoarcus-Aromatoleum* group and its separation into subgroups by different methods such as average nucleotide identity (ANI) and core genome analysis. A further 35 genomes from related genera (Table S1a) were also included in the analysis in order to place the *Azoarcus-Aromatoleum* group into a wider taxonomic/phylogenetic context.

For the purpose of genome sequencing, the following strains were obtained from various culture collections (in parentheses): *Az. communis* Swub3^T^ (DSM), *Az. communis* LMG 5514 (LMG), *Az. indigens* VB32^T^ (LMG), *Az. taiwanensis* NSC3 (LMG), *Ar. anaerobium* LuFRes1^T^ (DSM), *Ar. aromaticum* pCyN1 (DSM), *Ar. bremense* PbN1^T^ (DSM), *Ar. buckelii* U120^T^ (DSM), *Ar. diolicum* 22Lin^T^ (DSM), *Ar. evansii* KB740^T^ (DSM), *Ar. petrolei* ToN1^T^ (DSM), *Ar. toluolicum* T^T^ (DSM), and *Ar. toluvorans* Td21^T^ (DSM). In addition, the *Azoarcus* sp. strain TTM-91 was described for the first time, and its genome was also sequenced as part of this study. Strain TTM-91 was isolated on R2A agar from uncontaminated water sampled from the Caohu River (24°04′24″ N, 120°46′04″ E) in the vicinity of Taichung City, Taiwan.

The genomes of the 14 strains in Table S1b were sequenced by MicrobesNG (UK) with the genomic DNA library prepared using the Nextera XT Library Prep Kit (Illumina, San Diego, CA, USA) following the manufacturer’s protocol with the following modifications: 2 ng of DNA was used as input, and polymerase chain reaction (PCR) elongation time was increased to 1 min. DNA quantification and library preparation were carried out on a Microlab STAR automated liquid handling system (Hamilton, Birmingham, UK). Pooled libraries were quantified using the Kapa Biosystems Library Quantification Kit for Illumina on a Roche light cycler 96-qPCR machine (Roche, Basel, Switzerland). Libraries were sequenced on the Illumina HiSeq (Illumina) using a 250 bp paired end protocol. Read quality was verified with FastQC v0.11.5 [28] and reads were adapter trimmed and quality filtered using Trimmomatic v0.39 (http://www.usadellab.org/cms/index.php?page=trimmomatic) with a sliding window quality cut-off of Q15 [29]. Possible contaminations were removed with the bbsplit.sh script from BBMAP v37.87 [30] by removal of reads mapping to the contaminant’s genomes (e.g., human genome). De novo genome assembly was carried out with SPAdes v3.11.1 [31] and genes were annotated using Prokka v1.11 [32]. The assembly (contig) sizes were determined with QUAST v5.0.2 [33].

### 2.2. NCBI Sequence Datasets

All 67 (*Azoarcus-Aromatoleum* and related genera) genomes and nucleotide and amino acid coding sequences were downloaded from the NCBI GenBank in FASTA and GenBank formats and are presented in Table S1a.

### 2.3. Average Nucleotide Identity (ANI)

In order to relate our newly generated genomic resources to the pre-existing *Azoarcus-Aromatoleum* resources in the NCBI database, we applied whole genome comparisons by average nucleotide identity (ANI) using the alignment-free tool FastANI (version 1.31) [43] (available at: https://github.com/ParBLiSS/FastANI). We conducted two independent analyses: first, the pairwise calculation of the ANI values was performed with the 32 genomes listed in Table S2a; second, these calculations were performed with 52 genomes after the inclusion of 20 *Thauera* genomes (Table S2b). As a tool input requirement, the nucleotide coding sequences (CDS) were concatenated into a single multiFASTA. We performed the ANI pairwise values calculation for genomes using the FastANI default parameters. The FastANI output files (with a search summary and a lower triangular matrix containing the identity values in phylip format) were used to determine dissimilarity matrices and to construct a hierarchical neighbour-joining (NJ) tree cluster to reorder the lines and columns of the ANI matrix and construct a heat map. These analyses were performed using a Linux operating system (Ubuntu 20.4.1 LTS) and MATLAB^®^ support programming.

### 2.4. Protein Clusters

The complete set of proteins translated from the CDS of each of the 67 genomes were joined in a FASTA file and clustered using the RAFTS3G tool [44] (available at: https://sourceforge.net/projects/rafts-g/) applying a minimal self-score of 0.7 as a threshold parameter for a specific protein to be included in a cluster. Two groupings were performed separately, RAFTS3G-32 for the *Azoarcus-Aromatoleum* group and RAFTS3g-67 for the 67 genomes. For the *Azoarcus-Aromatoleum* group, from an input multiFASTA of 145,695 protein sequences, RAFTS3G generated 13,959 clusters with two or more proteins (89.68% of the sequences) and 15,043 clusters with only one protein (10.32% of the sequences). For the 67 genomes, from a multiFASTA with 271,578 protein sequences, RAFTS3G generated 19,666 clusters with two or more proteins (89.39% of the sequences) and 28,811 clusters with only one protein (10.61% of the sequences).

The protein clusters were the basis for several steps in the subsequent phylogenetic/taxonomic analyses including core genome determination, *nif* gene cluster analysis, partial SWeeP genome representations, and specific common genes analyses.

### 2.5. Core Genome Analysis Based on Core Protein Groups

The core genome in the *Azoarcus-Aromatoleum* group was extracted and 1044 gene clusters were found in common in all 32 genomes (25.08% of the sequences). The analysis of core genome for all 67 genomes revealed only 231 common gene clusters (6.91% of the sequences).

### 2.6. Analysis of Nif and Other Functional Genes

Functional analyses of the *nif* clusters and *nifH* gene were performed for all 67 genomes. Analyses of the denitrification pathway and of aromatic compound degradation were performed only for the *Azoarcus-Aromatoleum* group.

The RAFTS3G-67 clusters were used to extract *nif* genes and genes for denitrification and benzoate metabolism. In this study, *nif* cluster analyses were performed using a manually curated “in-house” database containing 1546 genes present in the *nif* gene clusters of 144 diazotrophic organisms (Table S1c). This is an expanded version based on the study by Dos Santos et al. [45]. After database searches and manual validation, all *nifH* genes were found in the same RAFTS3G-67 grouping.

Analysis of functional genes was performed for the nitrate reduction and the aromatic compound degradation pathways. For nitrate reduction pathway analysis, we searched the RAFTS3G-32 clusters to identify the presence of genes encoding nitrate reductase (*nar*/*nap*), nitrite reductase (*nir*), nitric oxide reductase (*nor*), and nitrous oxide reductase (*nos*) using *Aromatoleum* sp. CIB corresponding marker genes (RAFTS3, [44,46]) and curated them manually. Aromatic compound degradation pathway genes including those for the anaerobic benzoate degradation pathway gene cluster (*bzd*), aerobic benzoate degradation pathway gene cluster (*box*), and genes from the “lower pathway” (*LP*) as marker genes were similarly searched in the RAFTS3G-32 groups.

### 2.7. SWeeP Phylogenies

Phylogenetic analyses were conducted using SWeeP [47] for protein set representations (available at: https://sourceforge.net/projects/spacedwordsprojection/). The purpose of this method is to transform any set of amino acid sequences into a single vector that can, for example, represent all proteins in an organism. In practice, the input file to generate the SWeeP vectors consists of a multiFASTA file containing amino acid sequences, in which the protein sequences in a particular organism are concatenated, each protein flanked by delimiters, to get a single sequence [47].

SWeeP vectors based on the complete set of proteins for each of the 32 *Azoarcus-Aromatoleum* genomes and for the wider group of 67 genomes were obtained to represent complete genomes. The parameters used were for spaced words, the “11011” mask, and for the projection into a size of 1369 for visualisation (37 × 37, a perfect square number, is desired for some visualisation tasks). These parameters were the same as those used for complete bacterial genomes in the study by De Pierri et al. [47] (see Figure S1).

The NJ model based on Euclidean distance matrices of SWeeP vectors was used for four analyses: phylogenetic analyses of complete genomes, phylogenetic trees and heatmaps for the analysis of the core genome, *nif* cluster, and *nifH* gene analyses. All phylogenetic trees were built using standard MATLAB functions and scripts (for an excerpt of script, see File S1, visualised with Dendroscope (version 3.7.2) [48] (available at: https://software-ab.informatik.uni-tuebingen.de/download/dendroscope3/welcome.html) and arranged with Adobe Illustrator CC 2017 (version 21.0.0, Adobe Corporation, San Jose, CA, USA).

### 2.8. Heat Maps

The heatmap graphs were constructed using “in-house” MATLAB^®^ scripts and arranged using Adobe Illustrator CC 2017 (version 21.0.0). For the FastANI heatmap, the pairwise ANI values matrix was normalised by the mean values: First, ANI values were converted into dissimilarity matrices, then, the lower and upper matrices were divided by their corresponding mean values. It was decided to normalise the mean to guarantee a better correspondence between the matrices in the visualisation.

The results of FastANI were used to determine dissimilarities matrices to construct a hierarchical NJ tree cluster in which the order of lines and columns were established according to these clusters.

SWeeP vector heatmaps were generated from the distance matrices normalised by the average values, and the order of the rows and columns were determined by corresponding NJ-based dendrograms (see File S1). This technique allowed us to compare different distance/dissimilarity matrices in the same heat map.

### 2.9. 16S rRNA Analysis

For the 16S rRNA-based phylogeny, the genes were extracted automatically from 54 genomes from the NCBI GenBank using an “in-house” MATLAB^®^ script and are listed in Table S1a. From the SILVA database [49], 13 16S rRNA genes from *Dechloromonas* sp. strains H13, Dech2017 and CZR5, *D. hortensis* MA-1, *D. agitata* is5, *Thauera* sp. UPWRP, *T. butanivorans* NBRC 103042, *T. linaloolentis* 47Lol_1 and 47Lol_2, *T. hydrothermalis* GD-2, *T. phenolivorans* ZV1C, *Rhodocyclus tenuis* DSM109T, and *Rubrivivax gelatinosus* CBS were collected. The 16S rRNA gene phylogenetic analyses were performed using Clustal Ômega [50] with default parameters. The phylogenetic trees were arranged with Adobe Illustrator CC 2017 (version 21.0.0).

### 2.10. Nitrogenase Activity

The acetylene reduction assay was used to determine nitrogenase activity on free-living cultures growing on semi-solid or liquid media [51,52]. Growth conditions for all strains are detailed in the Supplementary Materials (File S2). Ethylene (C_2_H_4_) formation was determined after incubation of the cultures in acetylene by using a Perkin Elmer Clarus 480 gas chromatograph equipped with a HayeSep^®^ N (80–100 MESH) column (Merck Life Science Ltd., Dorset, UK). The injector and oven temperatures were kept at 100 °C, while the flame ionisation detector was set at 150 °C. The carrier gas (nitrogen) flow was set at 8–10 mL min^−1^. Nitrogenase activity is reported as nmol of C_2_H_4_ min^−1^ mg protein^−1^. The ethylene calibration curve was prepared from the chemical decomposition of ethephon (Merck Life Science Ltd., Dorset, UK) as described by Zhang and Wen [53]. Whole cell protein concentration was determined by the Bradford method [54] after lysis in 0.1 mM NaOH.

## 3. Results and Discussion

### 3.1. Whole-Genome Sequences and Identity of Azoarcus sp. Strain TTM-91

Features about the genomes of the 14 *Azoarcus-Aromatoleum* strains that were sequenced for this study are presented in Table 1 and Table S1a,b. The genomes of an additional 18 strains were obtained from the NCBI, and these are listed in Table 1 together with their original sources. *Azoarcus* sp. TTM-91 was the only strain that was isolated specifically for this study. The 16S rRNA sequence of *Azoarcus* sp. strain TTM-91 suggests that it is closely-related to *Az. indigens* (>99% similarity), and it also has a very similar genome size (5,393,782 bp) and G + C content (67.70%) to *Az. indigens* VB32^T^ (Table 1 and Table S1b). The genomes of the remaining strains sequenced for this study were relatively standard in their size (ranging from 4,227,546 for *Ar. buckelii* to 6,025,652 for *Ar. toluiolicum*) and G + C content (ranging from 62.36% for *Az. communis* Swub3^T^ to 67.70% for *Azoarcus* sp. TTM-91) in the wider context of the *Azoarcus-Aromatoleum* group (Table 1 and Table S1b).

### 3.2. Average Nucleotide Identity (ANI) and Core Genome Analysis

**Figure 1:**
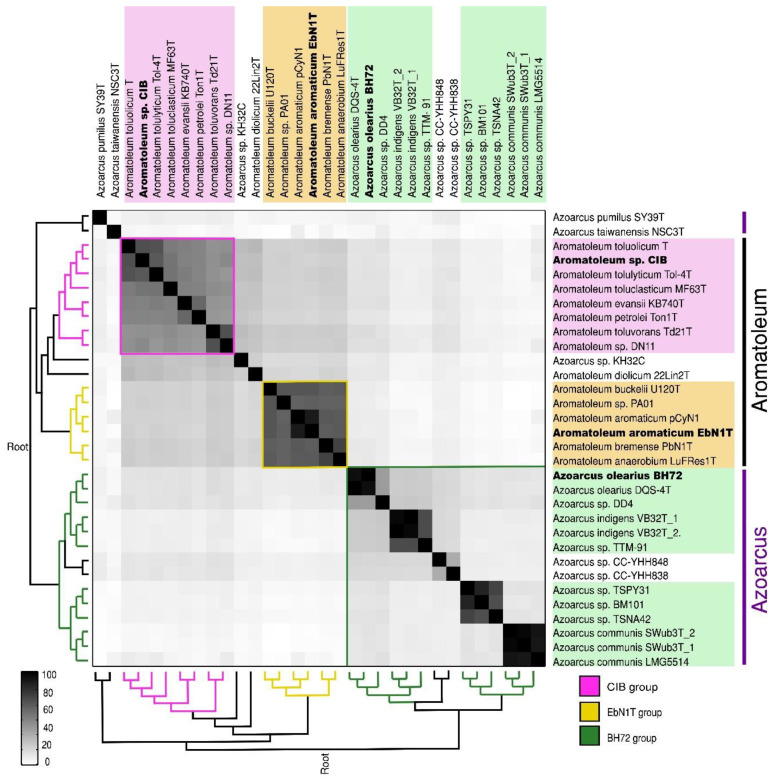
Average nucleotide identity (ANI) of whole genome comparison of 32 (18 database and 14 newly sequenced) *Azoarcus* and *Aromatoleum* genomes from 30 strains showing subgroups of the known genera *Azoarcus* and *Aromatoleum*. The depicted heatmap is a matrix generated by FastANI, ordered according to the dendrograms (left/below) obtained with the neighbour joining clustering method in MATLAB, considering upper and lower triangular complementary ANI dissimilarity matrices as input. All genome accession numbers are listed in Table 1 and Table S1a.

A matrix comparing the ANI of all 30 strains comprising 32 genomes (as two sequences were available for *Az. communis* Swub3^T^ and *Az. indigens* VB32^T^) is presented in Figure 1, and the ANI percentage values are given in Table S2a. The genomes of the 30 *Azoarcus-Aromatoleum* strains are broadly divided into the three main groups representing the genera *Azoarcus* and two large clades in *Aromatoleum*, but other groupings are also discernible. *Aromatoleum* contains two clearly separate groups: Group 1 (“EbN1 group”) comprising the species *Ar. anaerobium*, *Ar. aromaticum*, *Ar buckelii,* and *Ar. bremense*, together with the strain PAO1; and Group 2 (“CIB group”) comprising the species *Ar. diolicum, Ar. evansii, Ar. petrolei, Ar. toluclasticum, Ar. tolulyticum, Ar. toluolicum,* and *Ar. toluvorans,* together with the *“Azoarcus”* strains CIB and DN11 [20,37]. *Aromatoleum diolicum* sits just outside Group 2, and the strain KH32C appears not to belong to either group, but is slightly closer to the *Aromatoleum* Group 2 than to Group 1. In the ANI matrix, the genus *Azoarcus* is divided into “species complexes” represented by the type strains (plus any related genome-sequenced strains) of the three formally described species, *Az. communis*, *Az. indigens* (possibly including strain TTM-91 from the present study), and *Az. olearius*, plus a fourth, undescribed species related to *Az. communis* comprising two strains from South Korea (TSPY31, TSPN42) and the metagenome-derived strain BM101 from the USA. Outside these four well-defined, and related *Azoarcus* species complexes, are two Taiwanese strains “*Az. nasutitermitis*” CC-YHH838 and “*Az. rhizosphaerae*” CC-YHH848 [42], and the type strains of the single strain species *Az. pumilus* and “*Az. taiwanensis*”, which are distantly related to each other.

A two principal components projection for k-means clusters (k = 4) generated with SWeeP vectors demonstrated that the wider group of 67 genome-sequenced strains was clustered into four groups (Figure S2a), and these were linked with a core genome phylogeny (Figure S2b). This analysis allowed us to take a wider look at the *Azoarcus-Aromatoleum* group, particularly in the context of its neighbouring genus *Thauera*. It suggests that *Thauera* (or at least those 20 strains within it whose genomes have been fully sequenced) comprises two groups: a larger one in k3 consisting of 18 of the sequenced *Thauera* strains, and a much smaller subgroup containing only T. *hydrothermalis* GD-2T and *Thauera* sp. D20 that is placed in k2 (which also contains most of the *Azoarcus* strains). Both *Thauera* groups were more closely related to *Azoarcus* than to *Aromatoleum*, with *T. hydrothermalis* GD-2T and *Thauera* sp. D20 obviously nested within *Azoarcus*.

**Figure 2:**
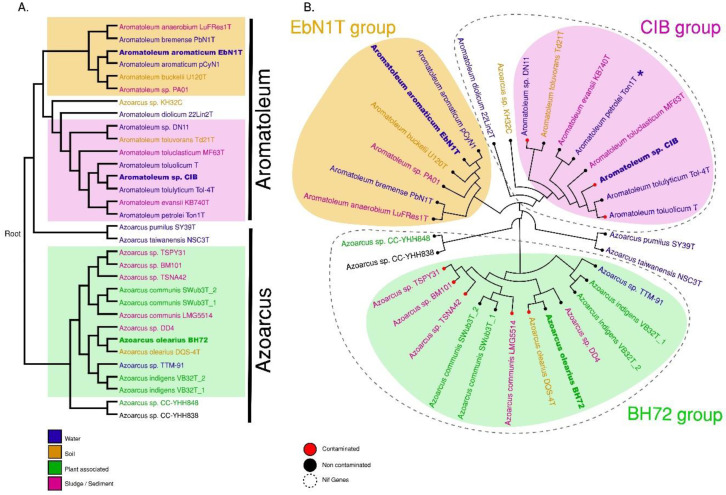
Phylogenetic trees of whole genomes (**A**) and clustering of core genomes (**B**). These analyses confirm the grouping of 30 *Azoarcus* and *Aromatoleum* strains (represented by 32 genomes) into three major groups named the EBN1, CIB, and BH72 groups. Both trees were constructed based on distance matrices of SWeeP vectors. The colours in the names indicate the source of the initial isolation of the corresponding strain: water (blue), soil (brown), sludge/sediment (pink) or plant-associated (green). Whether the substrate was contaminated (red) by organic solvents or non-contaminated (black) is indicated by the tip-point colour in the circular tree. The presence of *nif* genes in a group is indicated with dashed outlines. Asterisk (*) indicates the absence of *nif* genes in *Ar. petrolei* Ton1^T^.

A phylogenetic analysis was performed based on the whole genome and the core genome (Figure 2A,B). Three major groups can be discerned comprising (1) *Azoarcus* consisting of 14 strains including all currently accepted *Azoarcus* species plus the informally named *Az. nasutitermitis* (CC-YHH838), *Az. rhizosphaerae* (CC-YHH848) and *Az. taiwanensis*; (2) The “EbN1-Group” of six *Aromatoleum* strains including *Ar. aromaticum* EbN1^T^; and (3) the “CIB-Group” of 10 *Aromatoleum* strains plus “*Azoarcus*” sp. KH32C. Three small sub-groups are apparent in *Azoarcus*; the one closest to *Azoarcus sensu stricto* (*s.s.*) consists of two Taiwanese strains (CC-YHH838, CC-YHH848), and the other groups, which are more distant from *Azoarcus* (*s.s.*), consist of *Az. pumilus* and *Az. taiwanensis* from China and Taiwan, respectively. The EbN1-Group in *Aromatoleum* is monophyletic and could thus be termed *Aromatoleum sensu stricto* (*s.s.*), but the CIB-Group is looser and could be considered a polytomy (Figure 2A).

In terms of ecology/habitat, no obvious pattern could be discerned in the genotaxonomy of *Azoarcus-Aromatoleum* (Figure 2B) (i.e., the 30 taxa come from a wide range of habitats, and no habitat is particular to any of the three major groups). These include water from widely different sources (oceans, rivers, hot springs, aquifers, and wastewater treatment plants) as the primary habitat for the genome-sequenced *Azoarcus-Aromatoleum* strains, comprising 13 of the strains, sludge/sediment as the secondary habitat with eight strains, and soil as the tertiary habitat with four strains. Of the remaining five strains, all of which are in *Azoarcus*, one came from a termite nest in Taiwan (*Az. nasutitermitis* CC-YHH838), one from the rhizosphere of *Ficus religiosa* in Taiwan (*Az. rhizosphaerae* CC-YHH848), and three from roots of Kallar Grass including the model endophyte *Az. olearius* BH72, and the type strains of *Az. communis* (SwuB3^T^) and *Az. indigens* (VB32^T^). The genome-sequenced strains were also scored for habitats that were indicated as being contaminated by oil/petroleum; this showed that all the major groups in *Azoarcus-Aromatoleum* contained strains (seven of the genome-sequenced strains) that had an association with contaminated water or soils, but this association is by no means ubiquitous (Figure 2B).

In summary, although it should be stressed that the 30 strains presented in Figure 2B are only a “snapshot” of the habitats of *Azoarcus-Aromatoleum* strains, and their selection for our analysis is inevitably biased by the requirement for fully genome-sequenced strains, it is, nevertheless, clear from the present study and from perusing the databases for deposited gene sequences that water, sludge, and soil (contaminated and uncontaminated) are the principal habitats of all three *Azoarcus-Aromatoleum* groups. Plants are not a common habitat; only three strains in our analysis were actually isolated from plants (i.e., Kallar Grass), and all of these were from a single field in Pakistan [1,3]. Moreover, in each case, these plant-associated strains (*Az. olearius* BH72, *Az. communis* Swub3^T^, and *Az. indigens* VB32^T^) have sister strains in the same (or closely-related) species that were not isolated from plants such as *Az. olearius* DQS-4^T^ (oil-contaminated soil; [5]), *Az. communis* LMG 5514 (refinery oil sludge; [3]), and *Azoarcus* sp. TTM-91 (river water; this study). Other examples are *Azoarcus* sp. DD4, which is quite closely related to *Az. olearius* and may belong to this species (Figure 2A,B); this was isolated from activated sludge from a wastewater treatment plant [16]. Moreover, the other genome-sequenced strains in the *Az. communis* species complex (BM101, TSPY31, TSNA42), were isolated from sediments. Therefore, even though *Azoarcus* was the only group in the present study that contained plant-associated strains/species, these were few in number, and the most intensively studied among them, *Az. olearius* BH72, did not appear to possess genes that make it any more specialised in terms of associating with plants than its non-plant-associated sister *Az. olearius* DQS-4^T^ [5]. Therefore, widely used terms in the literature like “the plant-associated *Azoarcus* genus/group” (see Introduction) should henceforth be avoided as these give a misleading impression about the much wider habitat preferences of *Azoarcus*.

Phylogenetic whole genome analyses including *Thauera* (Figure S2a,b) also indicated that *T. hydrothermalis* GD-2^T^ and *Thauera* sp. D20 were actually nested within *Azoarcus*, and are apparently most closely related to the *Azoarcus* strains CC-YHH838 and CC-YHH848. *Thauera hydrothermalis* GD-2^T^ was isolated from the sediment of a hot spring in Tibet [55], and is a facultative anaerobe. Nothing has been published as yet about *Thauera* sp. D20, except that it was isolated from a saline lake in Inner Mongolia [56].

The analyses of whole genomes and of the core genomes of the 30 *Azoarcus-Aromatoleum* strains (Figure 2) has closely supported the groupings revealed by the ANI analysis. In addition, the taxonomy revealed by these genome-based analyses does not conflict with that shown by 16S rRNA sequences extracted from the genomes (Figure S3), which in itself is largely concordant with that of Rabus et al. [21], who used fewer (and mainly type) strains to construct their 16S rRNA phylogeny. The main differences between the whole genome analysis (Figures S2b and S4) and that based on 16S rRNA (Figure S3) is that the former places *T. hydrothermalis* GD-2^T^ and *Thauera* sp. D20 in *Azoarcus* (see above), but the latter places them in *Thauera* together with the *Azoarcus* strains CC-YHH838 and CC-YHH848 (Figure S3). Further studies including a polyphasic analysis should help determine if *T. hydrothermalis* GD-2^T^ and *Thauera* sp. D20 actually belong to *Azoarcus* or if they belong to *Thauera* together with CC-YHH838 and CC-YHH848.

At present, the delineation of bacterial species and, to some extent, genera is still based upon the 16S rRNA gene with suggested cut-offs of <97% similarity between related taxa for species, and a more arbitrary value of 94–95% for genera [57,58,59,60]. In addition, for generic delineations, it is recommended that phenotypic and lifestyle attributes be taken into account in addition to genetics. Accordingly, Rabus et al. [21] described the new genus *Aromatoleum* on the basis that they are facultative aerobes that can use nitrate as an alternative electron acceptor under anoxia, and that they have the ability to degrade aromatic compounds under anaerobic conditions. Although they also recognised that 16S rRNA sequences showed that *Aromatoleum* contained two clades corresponding to the Groups 1 (EbN1^T^) and 2 (CIB) described in the present study, Rabus et al. [21] recommended that in spite of this genetic separation that *Aromatoleum* should not be further divided as suggested by Martin-Moldes et al. [20], as all members of the new genus as delineated by them had the overarching and prominent property of anaerobic biodegradation of aromatic compounds. However, they did not include several strains that are pertinent to this debate such as *Azoarcus* sp. KH32C, *Az. pumilus,* and *Az. taiwanensis*, which fit into neither *Aromatoleum* nor *Azoarcus* as delineated by Rabus et al. [21]. Reinhold-Hurek et al. [17] described three new genera within *Azoarcus sensu lato* as it stood at that time (viz. *Azonexus*, *Azopira*, and *Azovibrio*), at least partly on the basis that their 16S rRNA sequences were only 93–94% similar to *Azoarcus* (*s.s.*). If 16S rRNA sequences were to be used in this manner to discern genera within the *Azoarcus-Aromatoleum* strains in the present study, it would suggest that *Azoarcus-Aromatoleum* constitutes a single genus with the exception of *Az. pumilus* and *Az. taiwanensis*, which would be placed into separate genera (Figure S3, Table S3a,b). However, we are rapidly moving into an era of genome-based taxonomies, and so 16S rRNA sequences are likely to be used less and less frequently as the sole gene sequence-based criterion for describing new taxa.

Although it has recently been demonstrated that ANI cannot be used for genus definition [61], it can still give indications of where generic boundaries might genuinely lie. In the present study, together with whole genome and core phylogenetics analysis from large numbers of genes, it is suggested that in terms of their core genomes, the two clades in *Aromatoleum* could constitute separate genera, but also that separate genera for *Az. pumilus* and *Az. taiwanensis* could be created, and possibly another for *Azoarcus* strain KH32C (Figures S2b and S4).

### 3.3. Genes for Nitrate Reduction (Nar/Nap, Nir, Nor, Nos) and for the Anaerobic (Bzd) or Aerobic (Box) Degradation of Benzoate

**Figure 3:**
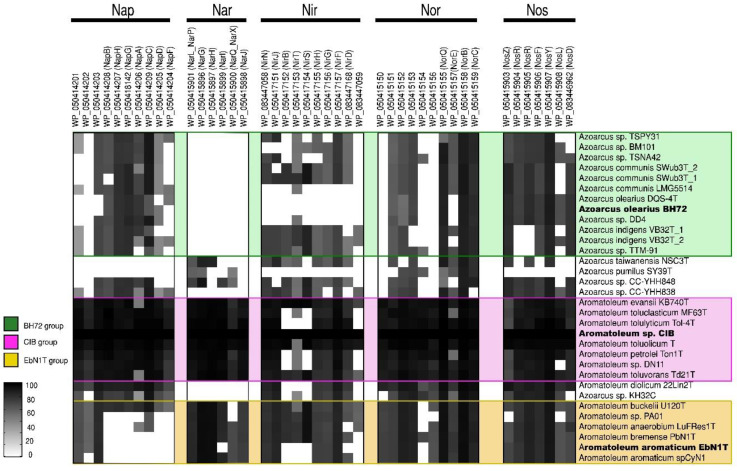
Nitrate reduction pathway analysis. Heatmap revealing the presence/absence of genes from the *nap*, *nar*, *nir*, *nor*, and *nos* gene clusters from 32 genomes (from 30 strains) of the *Azoarcus-Aromatoleum* group. The indicated marker genes were identified in the RAFTS3G-32 clusters containing corresponding genes in *Aromatoleum* sp. CIB and verified manually. The homology of the identified genes to reference genes is indicated with a grey scale gradient from white (0%) to black (100%).

**Figure 4:**
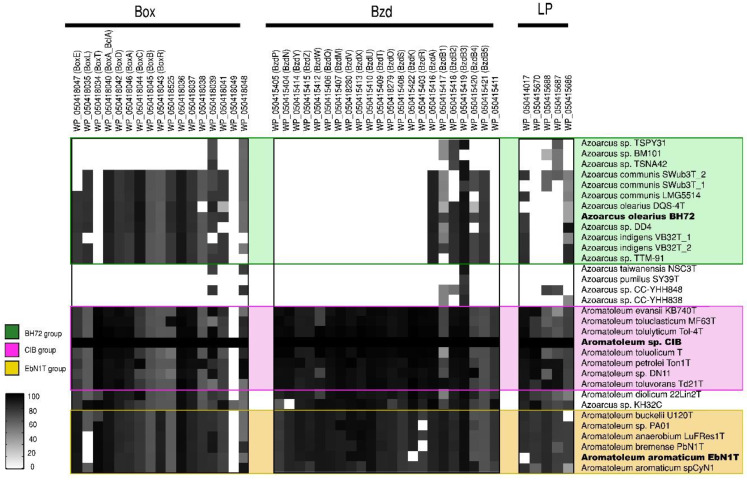
Aromatic compound degradation pathway analysis. Heatmap revealing the presence/absence of different genes from the benzoate (*bzd*) and benzoyl-coenzyme clusters (*box*) as well as of genes from the “lower pathway” (LP) present in 32 genomes (from 30 strains) of the *Azoarcus-Aromatoleum* group. The indicated marker genes were identified in the RAFTS3G-32 clusters containing corresponding genes in *Aromatoleum* sp. CIB and verified manually. The homology of the identified genes to reference genes is indicated with a grey scale gradient from white (0%) to black (100%).

Further insights into the taxonomy of *Azoarcus-Aromatoleum* were gained by comparing sets of genes involved in the use of nitrate as an alternative electron acceptor (*nar*/*nap*, *nir*, *nor*, and *nos*; [62]), thus allowing for anaerobic respiration (Figure 3) as well as genes involved in the anaerobic and aerobic catabolism of benzoate (*bzd* and *box,* respectively; [63,64]) (Figure 4). Using the genome of *Aromatoleum* sp. CIB as a reference (Figure 3), it was revealed that with the exception of *Az. pumilus* SY39^T^, all the genome-sequenced *Azoarcus* and *Aromatoleum* strains possessed genes for nitrate reduction (*nap* and/or *nar*), but *nar* appeared not to be present in the *Azoarcus* genomes, except for that of the strain CC-YHH848 and that of *Az. taiwanensis*. Moreover, although *Azoarcus* possessed *nap* genes, the *Aromatoleum* strains had a fuller complement of them; interestingly, strains in the CIB group were particularly well endowed with both *nap* and *nar* genes, whereas those in the EbN1 group (except for *Ar. buckelii*) were apparently lacking in key *nap* genes, and hence may be unable to utilize this pathway for nitrate reduction. The only other strains that lacked *nap* genes were *Az. pumilus* SY39^T^ and *Az. taiwanensis*. Other anomalous strains in terms of nitrate reduction were *Ar. diolicum* and *Azoarcus* sp. KH32C in that they were similar to *Azoarcus* s.s. in possessing *nap*, but not *nar* genes. All the examined genomes contained genes for nitrite reduction (*nir*), except for those of the three *Az. olearius* strains (DQS-4^T^, BH72, DD4), *Az. communis* LMG5514, and *Az. pumilus* SY39^T^ (Figure 3). It has long been known that BH72 does not possess nitrite reductase genes [40], but the present study suggests that this appears to be a characteristic of its species, *Az. olearius*. Of the key *nir* genes, *nirK* and *nirS* [62], the former was only identified in *Azoarcus* sp. KH32C. Moreover, although *nirS* was present in most, but not all of the genomes, it is currently unclear whether some of the strains that apparently lack both *nirS* and *nirK* (e.g., *Ar. aromaticum*, *Ar. petrolei*, *Ar. tolulyticum*, and *Ar. toluclasticum*) can actually reduce nitrite. Genes for the final two steps in denitrification, *nor* (nitric oxide reductase) and *nos* (nitrous oxide reductase), were present in all the sequenced *Azoarcus-Aromatoleum* genomes, except that *nos* genes could not be detected in *Az. pumilus* SY39^T^.

*bzd* genes were present in all of the *Aromatoleum* strains plus *Azoarcus* sp. KH32C (Figure 4), and some were also observed in *Az. olearius*, *Az. indigens,* and *Az. communis*, although it is unlikely that the latter has the capacity to metabolize benzoate under anaerobic conditions since the genes that encode for the key enzyme involved in the benzoyl-CoA reduction (*bzdNOPQ*) are absent in these strains. On the other hand, all the aforementioned strains also possess *box* genes, so it is probable that they might degrade benzoate aerobically [64], but the absence of *bzd* and *box* genes in the *Az. communis* strains TSPY31, TSPN42, and BM101 in *Az. pumilus* SY39^T^ and in *Az. taiwanensis* suggests that they cannot metabolize benzoate at all.

In summary, if the presence of *nap* and/or *nar* genes are indicative of a capacity to derive energy from respiratory nitrate reduction [62], then it is possible that all the *Azoarcus-Aromatoleum* strains in the present study possess it, with the exception of *Az. pumilus* SY39^T^. Indeed, Zamarro et al. [65] experimentally demonstrated nitrate reductase-based anaerobic metabolism for a modified variant of *Az. communis* Swub3^T^ with a *bzd* cassette inserted into its genome. In addition, it is also likely that all the strains, except for *Az. olearius*, *Az. communis* LMG5514, and *Az. pumilus* SY39^T^, have the genetic capacity to perform the complete denitrification of nitrate to N_2_.

### 3.4. Nif Genes and Nitrogenase Activity

**Figure 5:**
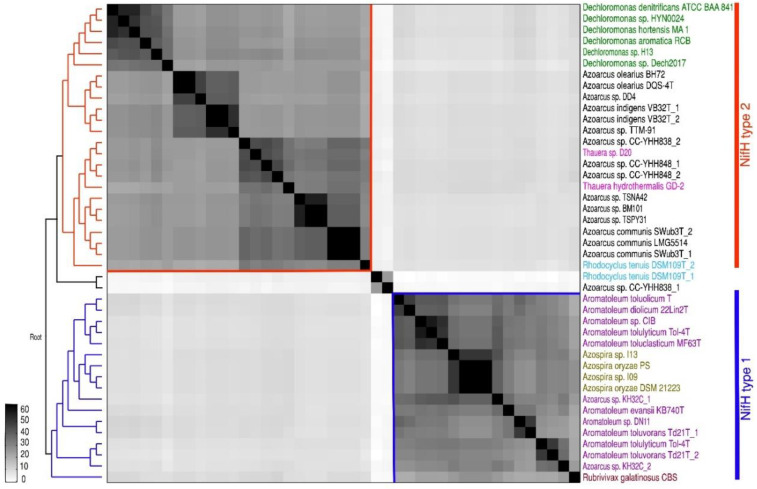
Single gene *nifH* phylogeny and heat map confirms the presence of two types of *nifH* genes in *Azoarcus-Aromatoleum* genomes. The *nifH* genes all belonged to a RATFTS3G-67 cluster that was identified by RAFTS3 searches and verified manually.

**Figure 6:**
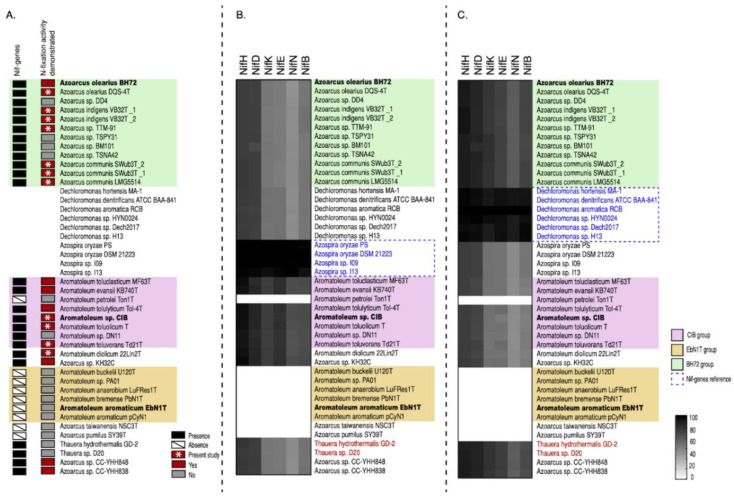
Analysis of the minimum set of nif genes for diazotrophy (*nifHDKENB*) reveals the presence of *Azospira*-type *nif* genes in the CIB-Group and *Dechloromonas*-type *nif* genes in the BH72-Group in *Azoarcus-Aromatoleum*. The presence of *nif* genes in black and experimentally proven nitrogenase (acetylene reduction) activity (ARA) in red are shown in panel (**A**) with ARA data obtained during the present study indicated by *. Known *nif* genes from the reference strains *Azospira oryzae* DSM 21223 (**B**) or *Dechloromonas aromatica* RCB (**C**) were used to identify homologous genes in the 32 analysed *Azoarcus* (green) and *Aromatoleum* (yellow and pink) genomes as well as in 20 related *Thauera* (note that only the two *Thauera* genomes harbouring *nif* genes are shown). For comparison, four *Azospira* (**A**) and six *Dechloromonas* (**B**) genomes are also included. The heatmaps indicate homology with a grey scale gradient from white (0%) to black (100%).

The presence of *nif* genes was analysed in all 30 *Azoarcus-Aromatoleum* genomes used in the present study as well as the related genera *Azospira*, *Dechloromonas,* and *Thauera* (Figure 5 and Figure 6, Figure S5; Table 1, Table S1a). The RAFTS3G-32 cluster analyses showed that *nifH* genes were present in 43 genomes in total. The following organisms lack the *nifH* gene and homology to all other *nif* genes (except *nif*US): *Ar. buckelii* U120^T^, *Aromatoleum* sp. PA01, *Ar. anaerobium* LuFRes1, *Ar. bremense* PbN1^T^, *Ar. aromaticum* EbN1^T^, *Ar. aromaticum* pCyN1, *Az. taiwanensis* NSC3^T^, and *Az. pumilus* SY39^T^. In contrast, two copies of the *nifH* gene were found in *Azoarcus* sp. CC YHH838, *Azoarcus* sp. CC YHH848, *Rhodocyclus tenuis* DSM109^T^, *Azoarcus* sp. KH32C, and *Ar. toluvorans* Td21^T^. All 12 *Azoarcus* strains contained the minimal set of *nif* genes necessary for nitrogen fixation (56) suggesting that all are diazotrophs including some not yet demonstrated experimentally for nitrogenase activity such as *Azoarcus* sp. strain DD4 (the nitrogenase activity of *Az. communis* LMG5514 and *Azoarcus* sp. TTM-91 was confirmed in the present study; Table 2). Interestingly, all members of the *Aromatoleum* CIB-Group, with the exception of *Ar. petrolei*, are diazotrophs, albeit with a very different *nif* gene complement to those in *Azoarcus*. This includes the recently described species *Ar. diolicum* and *Ar toluolicum*; both have been demonstrated to express nitrogenase activity in the present study (Table 2), and *Ar. diolicum* by Rabus et al. [21]. Although we were unable to demonstrate nitrogenase activity for *Ar. evansii* under any of the conditions tested (Table 2), the original description of this species as *Az. evansii* stated that activity could be detected [6], but did not give details about how the assays were performed. None of the *Aromatoleum* EbN1 Group possessed *nif* genes, as previously reported [12,21].

The potential origin of the *nif* genes as suggested by their closest relatives was investigated. On the basis of partial *nifD* and *nifH* gene sequences, Faoro et al. [5] suggested that these might be *Dechloromonas* for *Azoarcus* and *Azospira* for *Aromatoleum*. *Dechloromonas, Azospira, Azoarcus*, *Aromatoleum*, and *Thauera* are all in the order Rhodocyclales [18], but were in different families: *Dechloromonas* is in the Azonexaceae, *Azospira* in the Rhodocyclaceae, and *Azoarcus*, *Aromatoleum* and *Thauera* belong to the Zoogloeaceae. Phylogenies built from nearly full-length *nifH* (Figure 5) and from *nif* cluster genes (Figure S5) again confirmed the different *nif* origins of the two groups of diazotrophs in *Azoarcus-Aromatoleum*, and the relatedness of the *nif* cluster in the *Aromatoleum* CIB-Group to *Azospira* and that of *Azoarcus* to *Dechloromonas*. The minimal *nif* gene set required for nitrogen fixation (*nifH*, *nifD*, *nifK, nifE, nifN*, and *nifB*) [45] of *Azoarcus-Aromatoleum* were compared to *Azospira oryzae* 6a3T [17] and to *D. aromatica* strain RCBT [66] by RAFTS3G-32 cluster analyses. The heatmaps clearly show that the *nif* genes of the *Aromatoleum* CIB-Group strains are more closely related to *Azospira oryzae* (Figure 6B) and those of *Azoarcus* to *D. aromatica* (Figure 6C). They also illustrate the absence of *nif* genes in the *Aromatoleum* EbN1 Group and in *Thauera*. Interestingly, both the analyses using the single *nifH* phylogenies (Figure 5) and core *nif* genes (Figure 6) placed two *Thauera* strains (*T. hydrothermalis* GD-2T and *Thauera* sp. D20) within *Azoarcus*, and these were the same strains that were nested within *Azoarcus* in the whole-genome analysis (Figures S1 and S4). Rather than suggesting that the genus *Thauera* possessed some diazotrophic members, it lends support to our earlier contention that these “*Thauera*” strains should be more appropriately included within the genus *Azoarcus*.

Frequent gene duplications in the genome of *Azoarcus* sp. KH32C, *Ar. toluvorans* Td21T, and *Azoarcus* sp. CC-YHH848 were revealed by our analysis as well as the presence of two types of *nifH* genes in the genomes of *Azoarcus* sp. CC-YHH838 and *Rhodocyclus tenuis* DSM109^T^ (Figure 5).

The separate origins of the *nif* genes in *Azoarcus* (s.s.) and in *Aromatoleum* CIB Group (*Aromatoleum* Group 2) suggests that they were obtained from different sources via horizontal gene transfer (HGT). It is not clear when and why this happened. *Azospira oryzae*, the possible donor of *nif* to *Aromatoleum* Group 2 is a facultative anaerobe associated with Kallar Grass and rice with an ecology similar to *Azoarcus* spp. [17,67], whereas *Dechloromonas*, the possible donor of *nif* to *Azoarcus*, is also a facultative anaerobe, but is not normally found associated with plants [66] (i.e., the *nif* donors to the two groups of diazotrophs in *Azoarcus-Aromatoleum* have the opposite ecology to that which would have been intuitively expected). On the other hand, this makes more sense if the *nif* gene regulation in *Azoarcus* and *Aromatoleum* strains is compared (i.e., the *Az. olearius* BH72-related strains possess *nifL* and *nifA* linked to the *rnf1* gene cluster, whereas the *Aromatoleum* CIB group strains do not encode NifL, and in this case *nifA* is linked to *nifB*) (Figure S6). In contrast to the *Azoarcus* strains, the *Aromatoleum* CIB group strains encode a class of NifA proteins that possesses a cysteine-containing interdomain linker that confers oxygen sensitivity [68]. The more elaborate NifL-NifA regulatory system, when linked to the Rnf1 complex, which can support electron transfer to nitrogenase under aerobic conditions [69], is the one that is more likely to be associated with diazotrophic organisms that are more dependent on aerobic respiration such as *Azoarcus* [70]. This again illustrates how difficult it is to ascribe a definitive habitat, ecology, and lifestyle to any of the groups in *Azoarcus-Aromatoleum*. Good examples are the two *Az. olearius* strains, BH72 and DQS-4^T^, that were isolated, respectively, from plants and from oil-contaminated soil, and yet they are almost identical in terms of genes, putatively allowing them to have an endophytic lifestyle [5]. In addition, the former *Azoarcus* sp. strain CIB, which is now placed in *Aromatoleum* Group 2, a group of organisms not previously known to be associated with plants, contains many “plant-associated” features in its genome, and can associate with rice endophytically and even express nitrogenase (and possibly produce indole acetic acid) *in planta* [14,20,71].

## 4. Conclusions

The aim of this study was neither to describe nor to re-circumscribe new taxa within *Azoarcus-Aromatoleum*, but it does provide an opportunity to suggest how these new genomic data may be used for this purpose in future studies (combined with appropriate phenotypic criteria). The paraphyletic nature of the *Azoarcus-Aromatoleum* group revealed by the ANI analysis, and by phylogenetic analysis of the core genomes and the 16S rRNA sequences of 30 genome-sequenced strains suggests that its taxonomy could be revised solely on the basis of genetics to either (1) retain the generic name *Azoarcus* for its entirety, or (2) that if *Aromatoleum* is to be retained as a separate genus, it could be divided into two genera (the non-diazotrophic *Aromatoleum sensu stricto* and the mainly diazotrophic *Aromatoleum* Group 2 or “CIB-Group”), and three additional genera could be created comprising *Az. pumilus, Az. taiwanensis*, and the single strain lineage *Azoarcus* strain KH32C (which is so far undescribed to species level), respectively; thus creating six genera within *Azoarcus-Aromatoleum*. In terms of ecology, retaining the umbrella name *Azoarcus* might be justified, as with the exception of a few plant-associated strains in *Azoarcus* (s.s.), across the entire *Azoarcus-Aromatoleum* group as most strains/species are found in soil and water (often contaminated with petroleum or related compounds), sewage sludge, and seawater. On the other hand, if metabolism/lifestyle is considered to be the primary factor in describing bacterial genera, the ability of *Aromatoleum* to utilize nitrate as a terminal electron acceptor for the anaerobic degradation of aromatic compounds such as benzoate and the inability of *Azoarcus* (s.s.) to do this makes for an obvious division between *Azoarcus* and *Aromatoleum* as proposed by Rabus et al. [21]. However, it could be argued that if *Azoarcus* strains like *Az. communis* Swub3^T^ can be induced to perform the anaerobic degradation of benzoate by the relatively simple insertion of the *bzd* gene cassette [65], then even this phenotype is not so clear as a distinguishing feature. Additional complications are created by the presence of the aforementioned “intermediate” groups. *Azoarcus* sp. KH32C possesses the anaerobic benzoate degradation phenotype, but it clearly does not belong to *Aromatoleum*. Moreover, *Az. pumilus* and *Az. taiwanensis* are both facultative anaerobes [11,23], and neither possess *nif genes*, suggesting a closer affiliation to the EbN1 group of *Aromatoleum*, but genomically, they are not close to either *Azoarcus* or *Aromatoleum* (this study). Similarly, the *Azoarcus* strains CC-YHH838 and CC-YHH848 from Taiwan occupy a peripheral position within the genus *Azoarcus*, and their metabolism (aerobic vs. anaerobic) is so-far undescribed, but they do appear to have fairly typical *Azoarcus*-type *nif* genes (Figure 6).

Clearly, more work is required to resolve the taxonomy of *Azoarcus-Aromatoleum,* both in terms of sequencing more genomes, and in terms of examining lifestyles and habitats of this highly diverse and potentially useful group of micro-organisms.

## Figures and Tables

**Table 1:** Original sources of the strains used in phylogenies and evidence for diazotrophy.

Strain	Genome Size [bp]	Genome GC Content [%]	*Nif* Genes	Nitrogenase Activity Confirmed by Acetylene Reduction Assay (ARA)	Isolation Site and/or Type of Habitat	Country of Origin	Strain Origin and Description
*Aromatoleum anaerobium* LuFRes1^T^	4,482,959	66.72	No	No	Sewage sludge	Germany	[8]
*Aromatoleum aromaticum* EbN1^T^	4,727,255	64.68	No	No	River water	Germany	[12,21,34,35,36]
*Aromatoleum aromaticum* pCyN1	4,208,498	65.23	No	No	River water	Germany	[21]
*Aromatoleum bremense* PbN1^T^	4,319,217	65.57	No	No	River water	Germany	[21]
*Aromatoleum buckelii* U120^T^	4,227,546	65.33	No	No	Oxic soil	Germany	[10]
*Aromatoleum diolicum* 22Lin2^T^	5,138,050	64.22	Yes	Yes	River water	Germany	[21]
*Aromatoleum evansii* KB740^T^	5,883,543	65.98	Yes	Yes	Creek sediment	USA	[6]
*Aromatoleum petrolei* Ton1^T^	5,364,035	65.73	No	No	River water	Germany	[21]
*Aromatoleum* sp. CIB	5,257,030	65.84	Yes	Yes	Diesel fuel-contaminated aquifer	Switzerland	[20]
*Aromatoleum* sp. DN11	4,956,835	66.30	Yes	Yes	Gasoline-contaminated groundwater	Japan	[37]
*Aromatoleum* sp. PA01	3,908,240	66.10	No	No	Sewage sludge, wastewater treatment plant	Germany	[15]
*Aromatoleum toluclasticum* MF63^T^	5,925,983	66.00	No	No	Shallow aquifer sediment	USA	[9]
*Aromatoleum tolulyticum* Tol-4^T^	5,110,391	66.43	Yes	Yes	Petroleum contaminated aquifer sediments	USA	[7]
*Aromatoleum toluolicum* T^T^	6,025,652	65.93	Yes	Yes	Petroleum contaminated aquifer	Switzerland	[21]
*Aromatoleum toluvorans* Td21^T^	4,786,256	66.66	Yes	Yes	Muck soil	USA	[9]
*Azoarcus communis* LMG5514	4,996,403	62.45	Yes	Yes	Oily sludge, storage tank, petroleum refinery	France	[3]
*Azoarcus communis* SWub3^T^_1	4,976,326	62.36	Yes	Yes	Kallar Grass roots	Pakistan	[3]
*Azoarcus communis* SWub3^T^_2	5,004,685	62.50	Yes	Yes	Kallar Grass roots	Pakistan	[3,38]
*Azoarcus* sp. TSNA42	4,886,934	62.80	Yes	nt *	Oil-contaminated sediment	South Korea	[39]
*Azoarcus* sp. TSPY31	4,572,081	63.20	Yes	nt	Oil-contaminated sediment	South Korea	[39]
*Azoarcus* sp. TTM-91	5,393,782	67.70	Yes	Yes	River water	Taiwan	This study
*Azoarcus indigens* VB32^T^	5,562,509	67.31	Yes	Yes	Kallar Grass roots	Pakistan	[3]
*Azoarcus indigens* VB32^T^	5,464,470	67.60	Yes	Yes	Kallar Grass roots	Pakistan	[3]
*Azoarcus olearius* BH72	4,376,040	67.90	Yes	Yes	Kallar Grass roots	Pakistan	[3,40]
*Azoarcus olearius* DQS-4^T^	4,451,751	67.83	Yes	Yes	Oil-contaminated soil	Taiwan	[4,5]
*Azoarcus pumilus* SY39^T^	3,225,512	66.53	No	No	Sea water	China	[23]
*Azoarcus* sp. BM101	4,904,245	62.90	Yes	nt	Estuary sediment	USA	[41]
*Azoarcus* sp. CC-YHH838	4,723,750	67.50	Yes	Yes	Termite nest	Taiwan	[42]
*Azoarcus* sp. CC-YHH848	4,602,270	68.30	Yes	Yes	Rhizosphere of *Ficus religiosa*	Taiwan	[42]
*Azoarcus* sp. DD4	5,400,077	66.70	Yes	nt	Activated sludge, wastewater treatment plant	USA	[16]
*Azoarcus* sp. KH32C	5,818,755	65.03	Yes	Yes	Soil from field with paddy rice and soybean crops	Japan	[13]
*Azoarcus taiwanensis* NSC3^T^	4,228,584	62.88	No	No	Hot spring	Taiwan	[11]

* nt = not tested.

**Table 2:** Nitrogenase (acetylene reduction) activity of selected strains of *Azoarcus* and *Aromatoleum*.

Strain or Control	Activity ± SEM nmol C_2_H_4_ mg Protein^−1^ min^−1^	Conditions ^3^
Control ^1^	0.111 ± 0.088	SM-semi-solid
Control ^2^	not detected	SM-semi-solid
*Az. olearius* DQS-4^T^	23.222 ± 0.826	SM-semi-solid
*Az. communis* SWub3^T^	10.611 ± 0.778	SM-semi-solid
*Az. communis* LMG5514	26.025 ± 1.041	SM-semi-solid
*Az. indigens* VB32^T^	17.952 ± 0.653	1/2CVM-semi-solid
*Azoarcus* sp. TTM-91	3.960 ± 0.724	1/2CVM-semi-solid
*Aromatoleum* sp. CIB	1.163 ± 0.041	MNF + 3 mM Benzoate-liquid
*Ar. toluvorans* Td21^T^	1.318 ± 0.354	MNF + 3 mM Benzoate-liquid
*Ar. diolicum* 22Lin2^T^	0.479 ± 0.022	UMS + 30 mM Malate-liquid-1% O_2_
*Ar. toluolicum* T^T^	0.653 ± 0.049	UMS + 30 mM Malate-liquid-1% O_2_
*Ar. evansii* KB740^T^	not detected	All above conditions tested

^1^ DQS4 + 20 mM NH4Cl; ^2^ *nifA* deletion mutant derived from *Az. olearius* DQS-4T; ^3^ See Supplementary Materials for Details (File S2).

## Data Availability

The data presented in this study are available in the Supplementary Material.

## Supplementary Materials

The following are available online at https://www.mdpi.com/2073-4425/12/1/71/s1, File S1. SWeeP Analyses. File S2. Growth conditions for assessment of nitrogenase activity using the acetylene reduction assay. Table S1. Accession numbers of sequences used in phylogenies, abbreviations used, and original sources of strains (a), the genome sequencing statistics for strains sequenced in this study (b) and accession numbers of sequences used in the analysis of *nif* and other functional genes (c). Table S2. ANI percentage values for (a) the 32 genomes of *Azoarcus* and *Aromatoleum* strains and (b) the genomes of *Azoarcus*, *Aromatoleum* plus *Thauera* strains. Table S3. Percentage similarity values between 16S rRNA sequences extracted from genomes of (a) *Azoarcus* and *Aromatoleum* strains and (b) *Azoarcus, Aromatoleum*, *Thauera, Azospira, Dechloromonas, Zoogloea ramigera* NBRC 15342, *Rhodocyclus tenuis* DSM109^T^*,* and *Rubrivivax galatinosus* CBS strains. Figure S1. Clustering of *Azoarcus*, *Aromatoleum*, and *Thauera* (Branch 9468) in the global bacterial tree previously presented in the study by De Pierri et al. [47] (with 10,324 whole genomes), and available at: https://sourceforge.net/projects/spacedwordsprojection/. Figure S2. Whole genome comparison of 67 genomes analysed in this study based on SWeeP vectors. (a) Two principal components projection for k-means clusters (k = 4) generated with SWeeP vectors; k1, k2, k3, and k4 are related to groups in the neighbour joining phylogeny presented in (b). Figure S3. Clustal Omega phylogenetic Inference of 16S rRNA analysis for all 67 genomes in this study. Figure S4. Core gene cluster comparisons for all 67 genomes. As observed in the complete genome analysis, *Azospira* and *Dechloromonas* were separated from the other genomes in the NJ phylogeny based on SWeeP projection and the ordered vectors distance matrix heatmap. Figure S5. NJ phylogeny and heatmap illustrating the comparison of 67 genomes in this study based on SWeeP projection of the *nif* gene cluster set for each organism. Analogous relationships between *Aromatoleum-Azospira* and *Azoarcus-Dechloromonas* were observed, confirming the *nifH* gene analysis. Figure S6. Comparative analysis of the gene neighbourhoods of *nif* regulatory genes carried out with PATRIC 3.6.6 [72]. (A) Search conducted with NifL (ANQ83604.1) from *Azoarcus olearius* strain DQS4 as reference. *nifL*, its protein product and homologs are shown in red, with *nifA* homologs (labelled as either 2 or 12 according to product annotation) in green. *rnfA1, rnfB1*, and *rnfC1* are depicted in brown, yellow, and mauve, respectively. The gene depicted in light green (number 13) from *Azoarcus* sp.CC-YHH848 is also a homolog of *rnfC1*. The gene depicted in light blue at the left of the figure is *rnfD1*. (B) Search conducted with NifA (AKU10620.1) from *Aromatoleum* sp. CIB as reference. *nifA*, its protein product and homologs are shown in red. Genes shown in light green to the left of *nifA* encode hypothetical proteins. *nifB* is depicted in brown. Genes located to the right of *nifA*, depicted in dark blue (number 4) and cyan (number 6) are adjacent to *nifA* in seven of the strains shown and encode a nucleoside diphosphate kinase and a 23S RNA adenine methyl transferase, respectively.
