# Supplementary material for: Comparative Genomics Provides Insights into the Taxonomy of Azoarcus and Reveals Separate Origins of Nif Genes in the Proposed Azoarcus and Aromatoleum Genera
Source: Genes (Basel). 2021 Jan 7;12(1):71. doi: 10.3390/genes12010071 (PMC7825797; doi:10.3390/genes12010071)
Supplement: Supplementary file 1 [file genes-12-00071-s001.zip › genes-1027650-supplementary/genes-1027650-supplementary.pdf]

Supplementary materials Raitzz et al.

File S1. SweeP Analyses

In this study, not all results were performed on specific scripts. In some cases, the results were obtained step by step in the MATLAB® command window and in other data of interchange between the various applied software. Below, is included excerpts from scripts with details and the links to the tools used that can assist in understanding the methods.

1) Function to calculate the distance of SWeeP Vectors:

```
pdist()
```

pdist() is a standard function in MATLAB® that calculate the pairwise distance (by default Euclidean distances) between observations (vectors);

2) Excerpt of script to obtain neighbour joining:

```
treeout = seqneighjoin(xdist,'equivar',xnames);
```

Were *xdist* is a pdist vector (MATLAB standart result from a *pdist()* function call) and *xname* is a cell var with the nodes names.

3) Normalization by the mean of ANI matrices:

Here, the normalization was performed for the upper and lower ANI matrices separately (see the script below). First, ANI was converted into dissimilarity matrices and then the lower and upper matrices were divided by their corresponding mean values. It was decided to normalize the mean to guarantee a better correspondence between the matrices in the visualization. This technique allowed us to compare different distance / dissimilarity matrices in the same heat map.

```
% ANI matrix
MANI
% Transforms lower and upper ANI matrices in dissimilarity
matrices
```

```
D1 = 100 - tril(MANI)+tril(MANI)';
D2 = 100 - triu(MANI)+triu(MANI)'
% iani1 - Order for Lower triangular part of the ANI matrix
[~ iani1] = HFclus(distmat2pdist(D1),'Method','Nj');
% iani2 - Order for upper triangular part of the ANI matrix
[~ iani2] = HFclus(distmat2pdist(D2),'Method','Nj');
% HFclus is an 'in house' function used for several purposes; here it receives a MATLAB pdist vector and returns de order of the leaves in the corresponding neighbour joining phylogenetic tree (exploiting the MATLAB bioinformatics toolbox functions)

% Normalization and matrix reordering for Heat map
Mx = tril(D1)/mean(D1(:))+triu(D2)/mean(D2(:));
Mx = Mx(iani1,iani2);
% Transforms matrix into image
xHeat = mat2im3(Mx);
```

4) Links to all the programs used in this study:

FastQC

https://www.bioinformatics.babraham.ac.uk/projects/fastqc/

Trimmomatic

http://www.usadellab.org/cms/?page=trimmomatic

BBMap

https://sourceforge.net/projects/bbmap/

SPAdes

https://cab.spbu.ru/software/spades/

Prokka

https://github.com/tseemann/prokka

QUAST

http://quast.sourceforge.net/

FastANI

https://github.com/ParBLiSS/FastANI

RAFTS3G

https://sourceforge.net/projects/rafts-g/

SWeeP

https://sourceforge.net/projects/spacedwordsprojection/

Dendroscope

https://software-ab.informatik.uni tuebingen.de/download/dendroscope3/welcome.html

Clustal Omega

https://www.ebi.ac.uk/Tools/msa/clustalo/

IQ-Tree

http://www.iqtree.org/

File S2. *Growth conditions for assessment of nitrogenase activity using the acetylene reduction assay*

Azoarcus olearius DQS-4^T, *Az. communis* Swub3^T, *Az. communis* LMG 5514 (LMG), *Az. indigens* VB32^T, and *Azoarcus* sp. TTM-91 were grown on SM or CVM media, prepared following the composition described in [1]. The SM medium was supplemented with a vitamin solution (1 mg L^{-1} thiamine hydrochloride, 2 mg L^{-1} D-pantothenic acid, 1 mg L^{-1} nicotinic acid, and 0.1 mg L^{-1} biotin) modified from UMS medium [2]. Semi solid medium (SM or ½CVM) was prepared at the moment of use by mixing pre-warmed solid (1.5% agar) and liquid media to reach a final concentration of 0.18% agar. ½CVM is CVM [1] but with half of the amount of bactopeptone and yeast extract *i.e.* 1.5 and 0.5 g L^{-1}, respectively. *Aromatoleum* sp. CIB and *Ar. toluvorans* Td21^T were grown on MNF medium [3] supplemented with 3 mM sodium benzoate as carbon source.

Prior to the nitrogenase activity assay, *Az. olearius,* DQS-4^T, *Az. communis* Swub3^T, *Az. communis* LMG 5514 (LMG), *Az. indigens* VB32^T, and *Azoarcus* sp. TTM-91 were streaked from glycerol stocks into CVM amended with 20 mM NH_4Cl (CVM+N) and incubated at 30°C for 3–5 days. Isolated colonies were then inoculated into 3 mL of liquid CVM+N (in 30 mL plastic universal tubes) and incubated at 30°C and 120 rpm. Once the culture reached saturation, a further inoculation (1/100) in liquid medium was performed before the inoculation into semi-solid medium (SM amended with 0.5 mM sodium glutamate for *Az. olearius,* DQS-4^T, *Az. communis* Swub3^T, and *Az. communis* LMG 5514, or ½CVM for *Az. indigens* VB32^T, and *Azoarcus* sp. TTM-91). The semi-solid medium (3 mL) was set in a 7 mL glass vial (Fisherbrand™ #14873562). Liquid cultures (15–60 µL), pre-grown as above, were used for inoculation into the center-bottom of vials of semi-solid media which was aimed at achieving approximately the same number of cells (OD_{600nm} = 0.01 approximately) across the different inocula. Cells were incubated for 18–24 hours, or until the bacterial pellicle reached the meniscus. Once the pellicle was formed, the plastic cap from the vial was replaced by a suba-seal rubber stopper (n° 21) and the substrate, acetylene (10% of the gas phase), for the nitrogenase activity assay was injected. A similar pre-inoculation procedure was used to assay the nitrogenase activity in *Aromatoleum* sp. CIB and *Ar. toluvorans* Td21^T except that MNF medium was supplemented with 3 mM sodium benzoate as carbon source and 5 mM KNO_3 as nitrogen source. The second re-inoculation into liquid medium was performed on a higher volume, 20 mL of medium in a 100 mL Erlenmeyer flask at 30°C and 120 rpm. Cells were then washed in MNF + 3 mM sodium benzoate to remove excess nitrate and then re-inoculated into fresh medium without nitrate to an OD_{600nm} of approximately 0.01. Ten mL cells (OD_{600nm} of 0.01), were transferred to 20 mL glass universal tubes, sealed with suba-seal rubber stopper n°41 and incubated for 24 hours at 30°C and 120 rpm before injection of the acetylene (10% of the gas phase). *Ar. diolicum* 22Lin^T was grown on UMS medium supplemented with 30 mM malate as carbon source and 20 mM NH_4Cl. Prior to the acetylene reduction assay, 50 mL cells were collected by centrifugation (4000 rpm, 8 min) and re-suspended to an initial O.D600 of 0.4 (20 mL in an 100 mL Pyrex Erlenmeyer flask) in UMS plus 30 mM malate without added ammonium. The flask was stoppered with a suba-seal (nº 37) and the internal flask atmosphere was adjusted to approximately 1% O2. After 6 hours incubation, the acetylene reduction assay was performed as above.

References

1. Krause, A.; Julich, H.; Mankar, M.; Reinhold-Hurek, B. The regulatory network controlling ethanol-induced expression of alcohol dehydrogenase in the endophyte *Azoarcus* sp. strain BH72. *Mol. Plant-Microbe Interact.* **2017**, 30, 778-785, doi:MPMI-01-17-0013-R.

2. Pini, F.; East, A.K.; Appia-Ayme, C.; Tomek, J.; Karunakaran, R.; Mendoza-Suarez, M.; Edwards, A.; Terpolilli, J.; Rowoth, J.; Downie, J.A.; et al. Bacterial biosensors for in vivo

spatiotemporal mapping of root secretion. *Plant Physiol.* **2017**, 174, 1289-1306, doi:10.1104/pp.16.01302

3. Fernández, H.; Prandoni, N.; Fernández-Pascual, M.; Fajardo, S.; Morcillo, C.; Díaz, E.; Carmona, M. Azoarcus sp. CIB, an anaerobic biodegrader of aromatic compounds shows an endophytic lifestyle. *PLoS One* **2014**, *9*, e110771, doi:10.1371/journal.pone.0110771

S1.

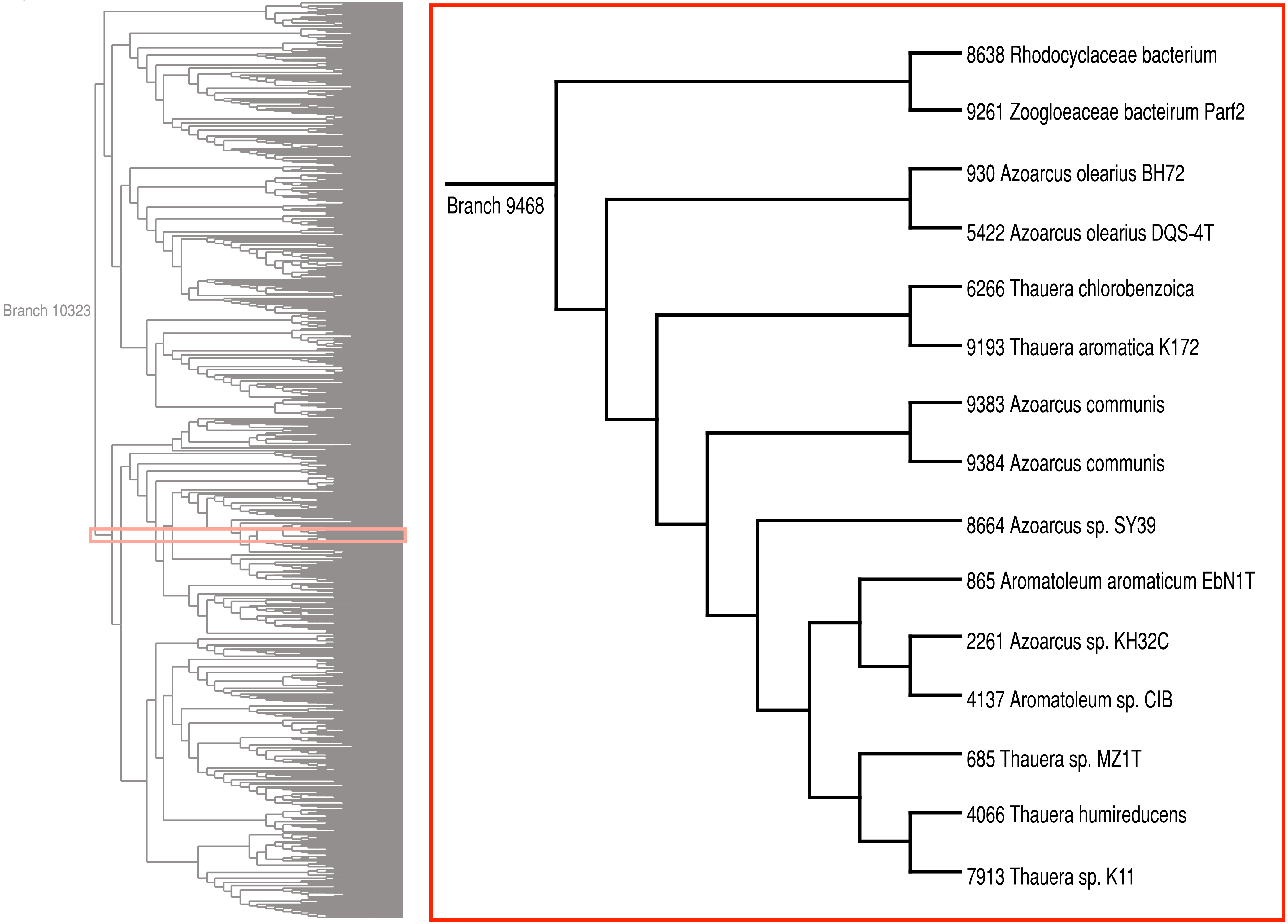
Branch 10323
Branch 9468
8638 Rhodocyclaceae bacterium
9261 Zoogloeaceae bacteirum Parf2
930 Azoarcus olearius BH72
5422 Azoarcus olearius DQS-4T
6266 Thauera chlorobenzoica
9193 Thauera aromatica K172
9383 Azoarcus communis
9384 Azoarcus communis
8664 Azoarcus sp. SY39
865 Aromatoleum aromaticum EbN1T
2261 Azoarcus sp. KH32C
4137 Aromatoleum sp. CIB
685 Thauera sp. MZ1T
4066 Thauera humireducens
7913 Thauera sp. K11

S2a.

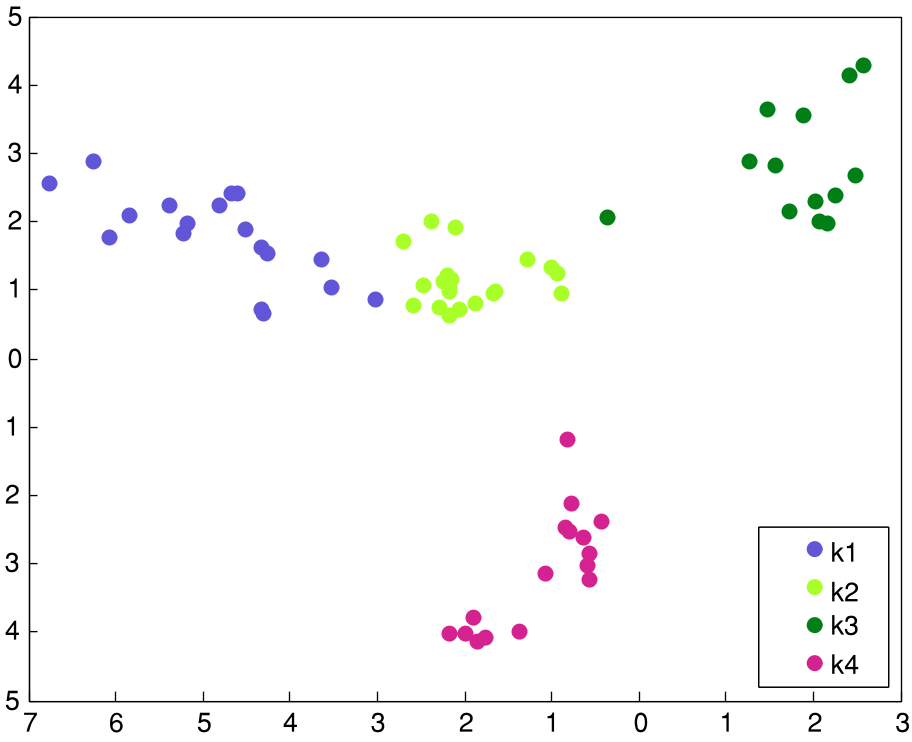

S2b.

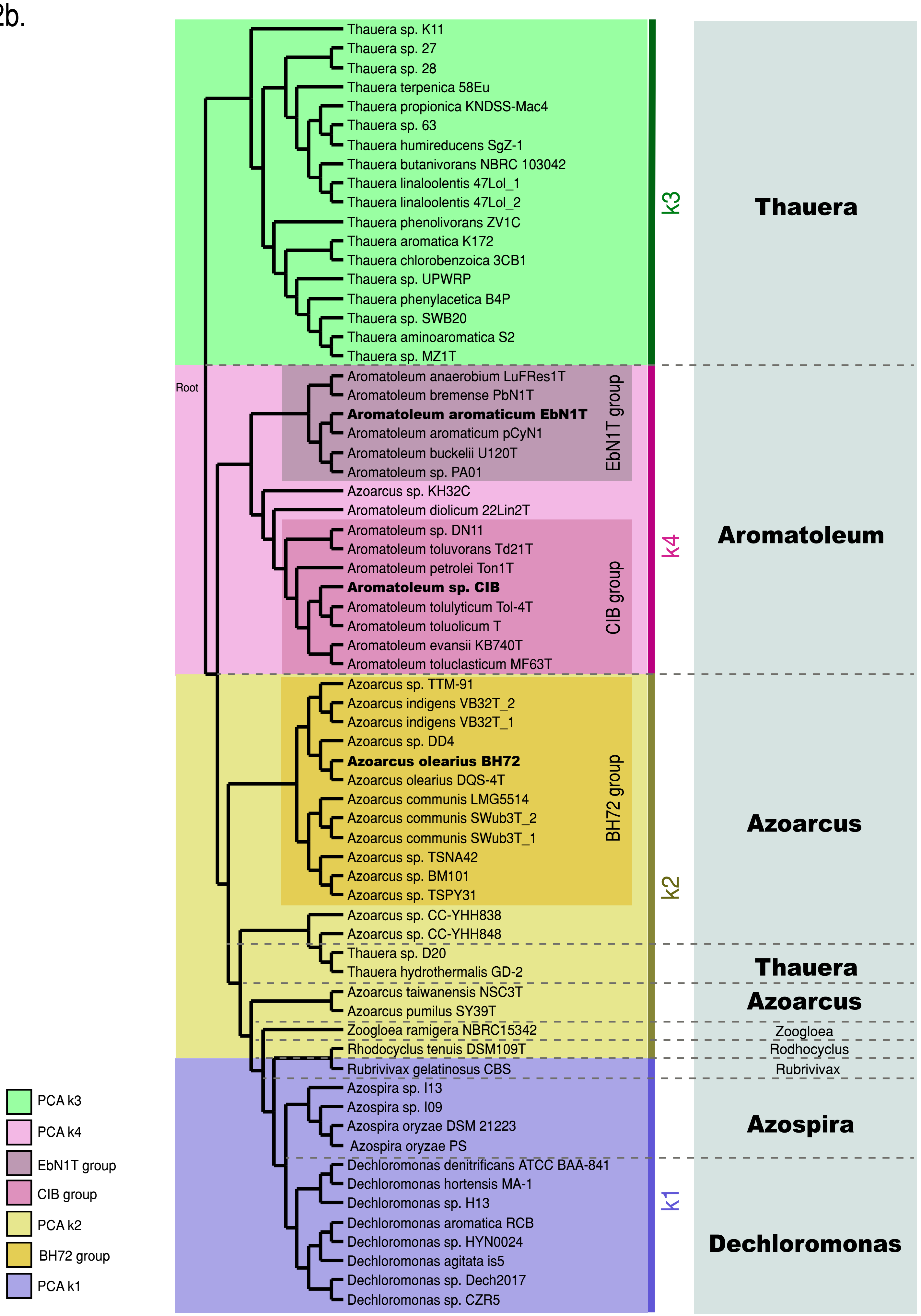

Thauera sp. K11
Thauera sp. 27
Thauera sp. 28
Thauera terpenica 58Eu
Thauera propionica KNDSS-Mac4
Thauera sp. 63
Thauera humireducens SgZ-1
Thauera butanivorans NBRC 103042
Thauera linaloolentis 47Lol_1
Thauera linaloolentis 47Lol_2
Thauera phenolivorans ZV1C
Thauera aromatica K172
Thauera chlorobenzoica 3CB1
Thauera sp. UPWRP
Thauera phenylacetica B4P
Thauera sp. SWB20
Thauera aminoaromatica S2
Thauera sp. MZ1T
Root
Aromatoleum anaerobium LuFRes1T
Aromatoleum bremense PbN1T
Aromatoleum aromaticum EbN1T
Aromatoleum aromaticum pCyN1
Aromatoleum buckelii U120T
Aromatoleum sp. PA01
EbN1T group
Azoarcus sp. KH32C
Aromatoleum diolicum 22Lin2T
Aromatoleum sp. DN11
Aromatoleum toluvorans Td21T
Aromatoleum petrolei Ton1T
Aromatoleum sp. CIB
Aromatoleum tolulyticum Tol-4T
Aromatoleum toluolicum T
Aromatoleum evansii KB740T
Aromatoleum toluclasticum MF63T
CIB group
Azoarcus sp. TTM-91
Azoarcus indigens VB32T_2
Azoarcus indigens VB32T_1
Azoarcus sp. DD4
Azoarcus olearius BH72
Azoarcus olearius DQS-4T
Azoarcus communis LMG5514
Azoarcus communis SWub3T_2
Azoarcus communis SWub3T_1
Azoarcus sp. TSNA42
Azoarcus sp. BM101
Azoarcus sp. TSPY31
BH72 group
Azoarcus sp. CC-YHH838
Azoarcus sp. CC-YHH848
Thauera sp. D20
Thauera hydrothermalis GD-2
Azoarcus taiwanensis NSC3T
Azoarcus pumilus SY39T
Zoogloea ramigera NBRC15342
Rhodocyclus tenuis DSM109T
Rubrivivax gelatinosus CBS
Azospira sp. I13
Azospira sp. I09
Azospira oryzae DSM 21223
Azospira oryzae PS
Dechloromonas denitrificans ATCC BAA-841
Dechloromonas hortensis MA-1
Dechloromonas sp. H13
Dechloromonas aromatica RCB
Dechloromonas sp. HYN0024
Dechloromonas agitata is5
Dechloromonas sp. Dech2017
Dechloromonas sp. CZR5
k3
k4
k2
k1
Thauera
Aromatoleum
Azoarcus
Thauera
Azoarcus
Zoogloea
Rodhocyclus
Rubrivivax
Azospira
Dechloromonas
PCA k3
PCA k4
EbN1T group
CIB group
PCA k2
BH72 group
PCA k1

S3

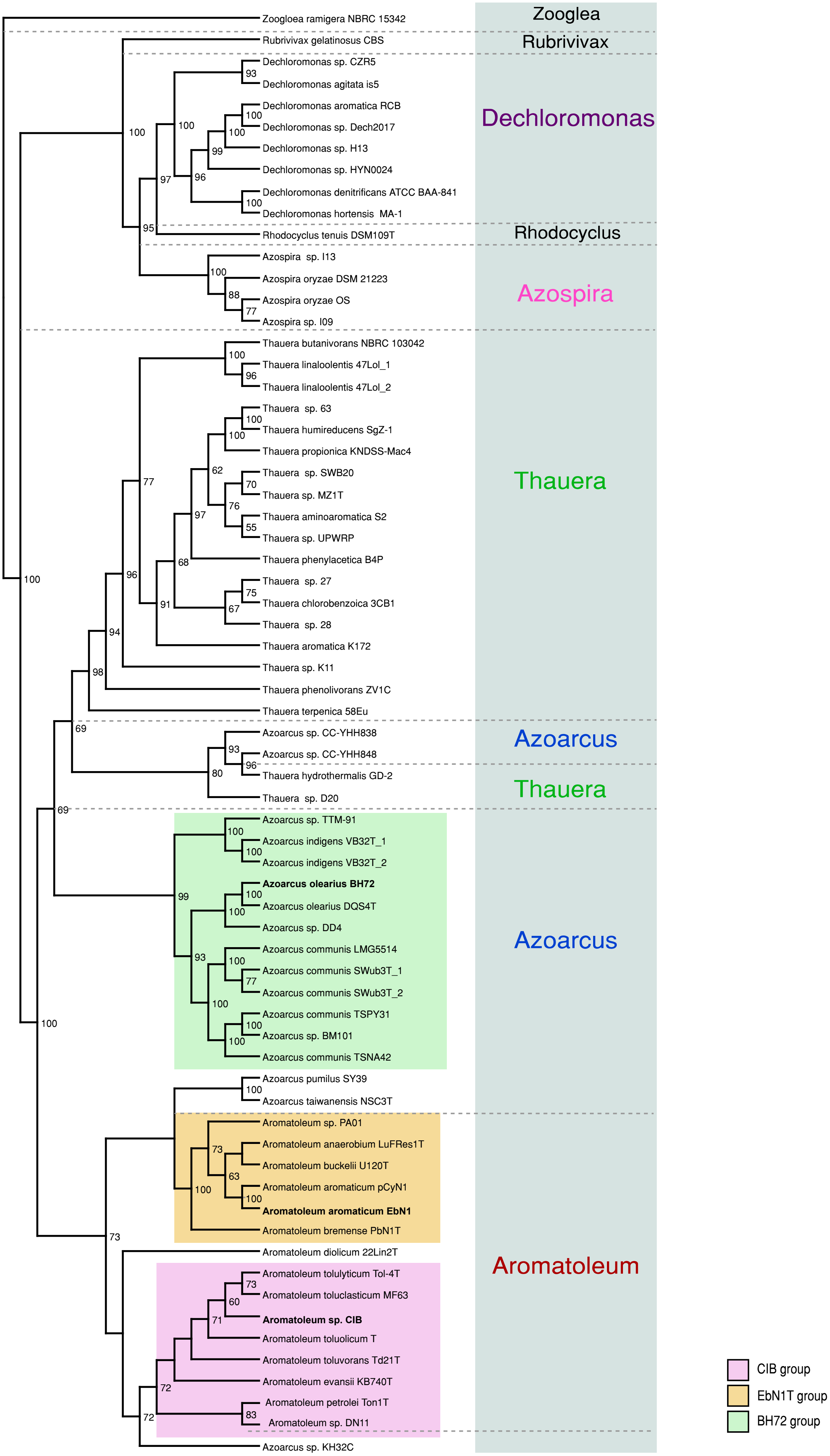
Zooglea
Zoogloea ramigera NBRC 15342
Rubrivivax
Rubrivivax gelatinosus CBS
Dechloromonas
Dechloromonas sp. CZR5
Dechloromonas agitata is5
Dechloromonas aromatica RCB
Dechloromonas sp. Dech2017
Dechloromonas sp. H13
Dechloromonas sp. HYN0024
Dechloromonas denitrificans ATCC BAA-841
Dechloromonas hortensis MA-1
Rhodocyclus
Rhodocyclus tenuis DSM109T
Azospira
Azospira sp. I13
Azospira oryzae DSM 21223
Azospira oryzae OS
Azospira sp. I09
Thauera
Thauera butanivorans NBRC 103042
Thauera linaloolentis 47Lol_1
Thauera linaloolentis 47Lol_2
Thauera sp. 63
Thauera humireducens SgZ-1
Thauera propionica KNDSS-Mac4
Thauera sp. SWB20
Thauera sp. MZ1T
Thauera aminoaromatica S2
Thauera sp. UPWRP
Thauera phenylacetica B4P
Thauera sp. 27
Thauera chlorobenzoica 3CB1
Thauera sp. 28
Thauera aromatica K172
Thauera sp. K11
Thauera phenolivorans ZV1C
Thauera terpenica 58Eu
Azoarcus
Azoarcus sp. CC-YHH838
Azoarcus sp. CC-YHH848
Thauera
Thauera hydrothermalis GD-2
Thauera sp. D20
Azoarcus
Azoarcus sp. TTM-91
Azoarcus indigens VB32T_1
Azoarcus indigens VB32T_2
Azoarcus olearius BH72
Azoarcus olearius DQS4T
Azoarcus sp. DD4
Azoarcus communis LMG5514
Azoarcus communis SWub3T_1
Azoarcus communis SWub3T_2
Azoarcus communis TSPY31
Azoarcus sp. BM101
Azoarcus communis TSNA42
Azoarcus pumilus SY39
Azoarcus taiwanensis NSC3T
Aromatoleum
Aromatoleum sp. PA01
Aromatoleum anaerobium LuFRes1T
Aromatoleum buckelii U120T
Aromatoleum aromaticum pCyN1
Aromatoleum aromaticum EbN1
Aromatoleum bremense PbN1T
Aromatoleum diolicum 22Lin2T
Aromatoleum tolulyticum Tol-4T
Aromatoleum toluclasticum MF63
Aromatoleum sp. CIB
Aromatoleum toluolicum T
Aromatoleum toluvorans Td21T
Aromatoleum evansii KB740T
Aromatoleum petrolei Ton1T
Aromatoleum sp. DN11
Azoarcus sp. KH32C
CIB group
EbN1T group
BH72 group

S4.

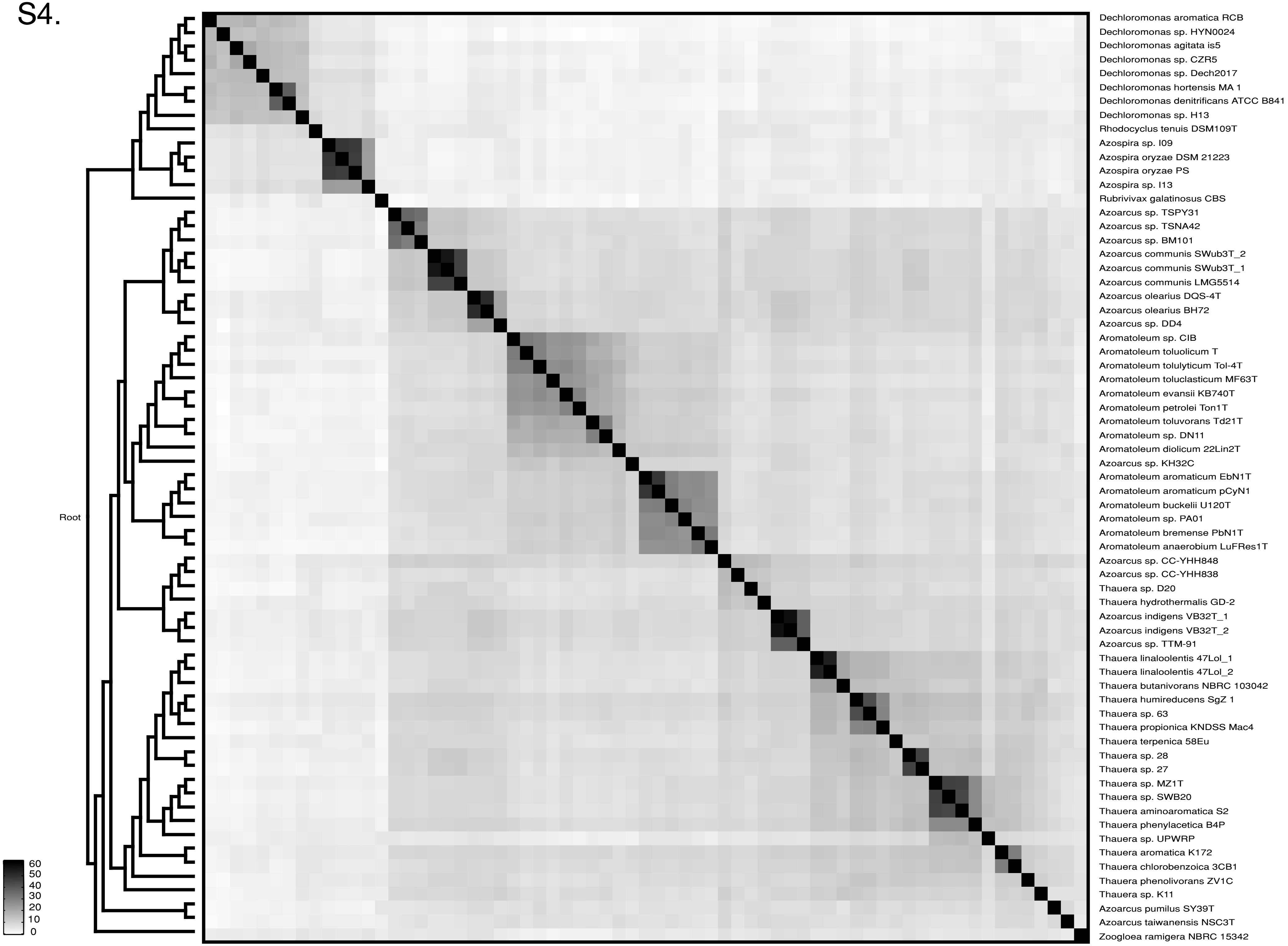
Root
60
50
40
30
20
10
0
Dechloromonas aromatica RCB
Dechloromonas sp. HYN0024
Dechloromonas agitata is5
Dechloromonas sp. CZR5
Dechloromonas sp. Dech2017
Dechloromonas hortensis MA 1
Dechloromonas denitrificans ATCC B841
Dechloromonas sp. H13
Rhodocyclus tenuis DSM109T
Azospira sp. I09
Azospira oryzae DSM 21223
Azospira oryzae PS
Azospira sp. I13
Rubrivivax galatinosus CBS
Azoarcus sp. TSPY31
Azoarcus sp. TSNA42
Azoarcus sp. BM101
Azoarcus communis SWub3T_2
Azoarcus communis SWub3T_1
Azoarcus communis LMG5514
Azoarcus olearius DQS-4T
Azoarcus olearius BH72
Azoarcus sp. DD4
Aromatoleum sp. CIB
Aromatoleum toluolicum T
Aromatoleum tolulyticum Tol-4T
Aromatoleum toluclasticum MF63T
Aromatoleum evansii KB740T
Aromatoleum petrolei Ton1T
Aromatoleum toluvorans Td21T
Aromatoleum sp. DN11
Aromatoleum diolicum 22Lin2T
Azoarcus sp. KH32C
Aromatoleum aromaticum EbN1T
Aromatoleum aromaticum pCyN1
Aromatoleum buckelii U120T
Aromatoleum sp. PA01
Aromatoleum bremense PbN1T
Aromatoleum anaerobium LuFRes1T
Azoarcus sp. CC-YHH848
Azoarcus sp. CC-YHH838
Thauera sp. D20
Thauera hydrothermalis GD-2
Azoarcus indigens VB32T_1
Azoarcus indigens VB32T_2
Azoarcus sp. TTM-91
Thauera linaloolentis 47Lol_1
Thauera linaloolentis 47Lol_2
Thauera butanivorans NBRC 103042
Thauera humireducens SgZ 1
Thauera sp. 63
Thauera propionica KNDSS Mac4
Thauera terpenica 58Eu
Thauera sp. 28
Thauera sp. 27
Thauera sp. MZ1T
Thauera sp. SWB20
Thauera aminoaromatica S2
Thauera phenylacetica B4P
Thauera sp. UPWRP
Thauera aromatica K172
Thauera chlorobenzoica 3CB1
Thauera phenolivorans ZV1C
Thauera sp. K11
Azoarcus pumilus SY39T
Azoarcus taiwanensis NSC3T
Zoogloea ramigera NBRC 15342

S5.

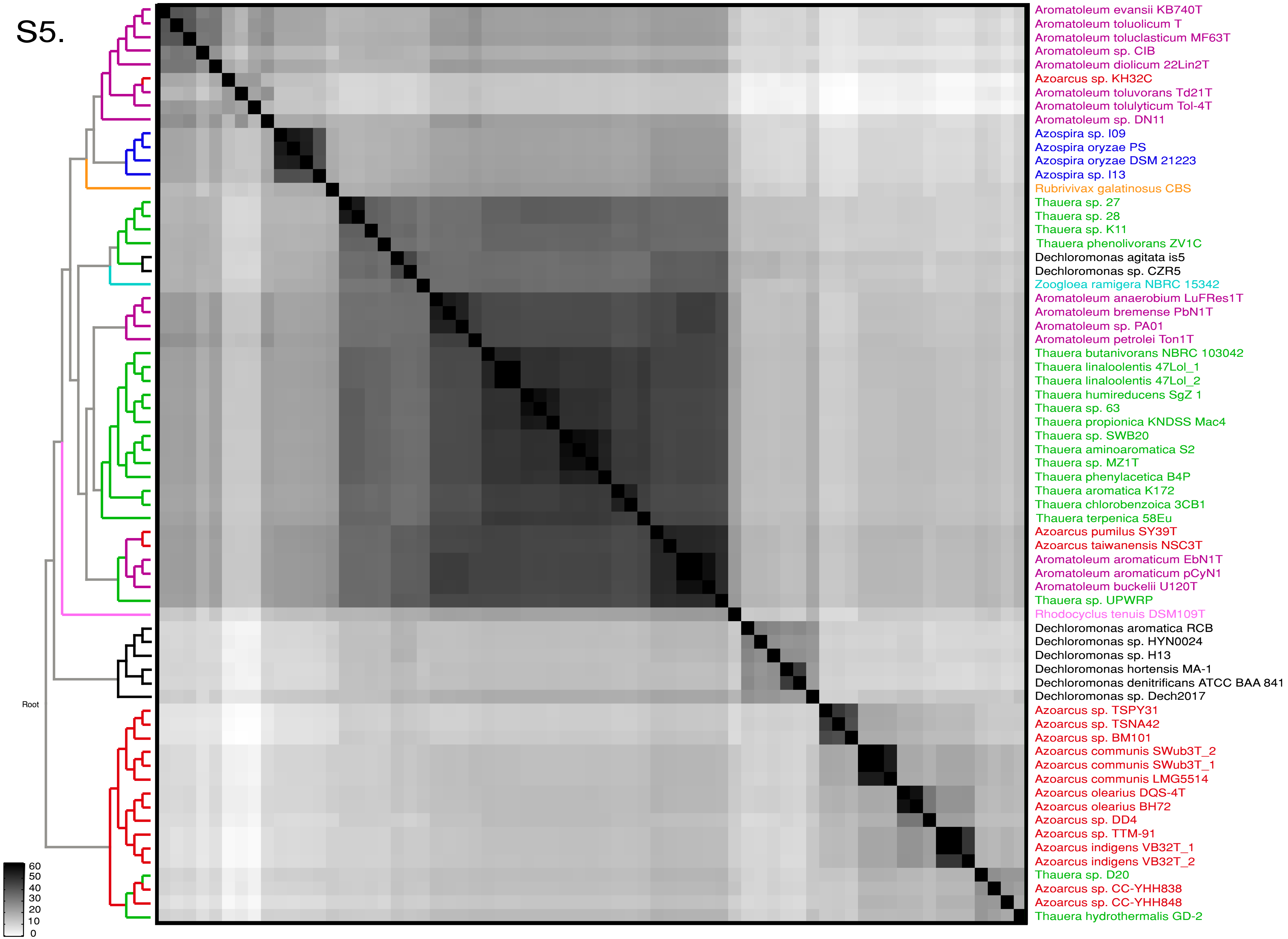
Aromatoleum evansii KB740T
Aromatoleum toluolicum T
Aromatoleum toluclasticum MF63T
Aromatoleum sp. CIB
Aromatoleum diolicum 22Lin2T
Azoarcus sp. KH32C
Aromatoleum toluvorans Td21T
Aromatoleum tolulyticum Tol-4T
Aromatoleum sp. DN11
Azospira sp. I09
Azospira oryzae PS
Azospira oryzae DSM 21223
Azospira sp. I13
Rubrivivax galatinosus CBS
Thauera sp. 27
Thauera sp. 28
Thauera sp. K11
Thauera phenolivorans ZV1C
Dechloromonas agitata is5
Dechloromonas sp. CZR5
Zoogloea ramigera NBRC 15342
Aromatoleum anaerobium LuFRes1T
Aromatoleum bremense PbN1T
Aromatoleum sp. PA01
Aromatoleum petrolei Ton1T
Thauera butanivorans NBRC 103042
Thauera linaloolentis 47Lol_1
Thauera linaloolentis 47Lol_2
Thauera humireducens SgZ 1
Thauera sp. 63
Thauera propionica KNDSS Mac4
Thauera sp. SWB20
Thauera aminoaromatica S2
Thauera sp. MZ1T
Thauera phenylacetica B4P
Thauera aromatica K172
Thauera chlorobenzoica 3CB1
Thauera terpenica 58Eu
Azoarcus pumilus SY39T
Azoarcus taiwanensis NSC3T
Aromatoleum aromaticum EbN1T
Aromatoleum aromaticum pCyN1
Aromatoleum buckelii U120T
Thauera sp. UPWRP
Rhodocyclus tenuis DSM109T
Dechloromonas aromatica RCB
Dechloromonas sp. HYN0024
Dechloromonas sp. H13
Dechloromonas hortensis MA-1
Dechloromonas denitrificans ATCC BAA 841
Dechloromonas sp. Dech2017
Azoarcus sp. TSPY31
Azoarcus sp. TSNA42
Azoarcus sp. BM101
Azoarcus communis SWub3T_2
Azoarcus communis SWub3T_1
Azoarcus communis LMG5514
Azoarcus olearius DQS-4T
Azoarcus olearius BH72
Azoarcus sp. DD4
Azoarcus sp. TTM-91
Azoarcus indigens VB32T_1
Azoarcus indigens VB32T_2
Thauera sp. D20
Azoarcus sp. CC-YHH838
Azoarcus sp. CC-YHH848
Thauera hydrothermalis GD-2
Root
60
50
40
30
20
10
0

S6.

A.

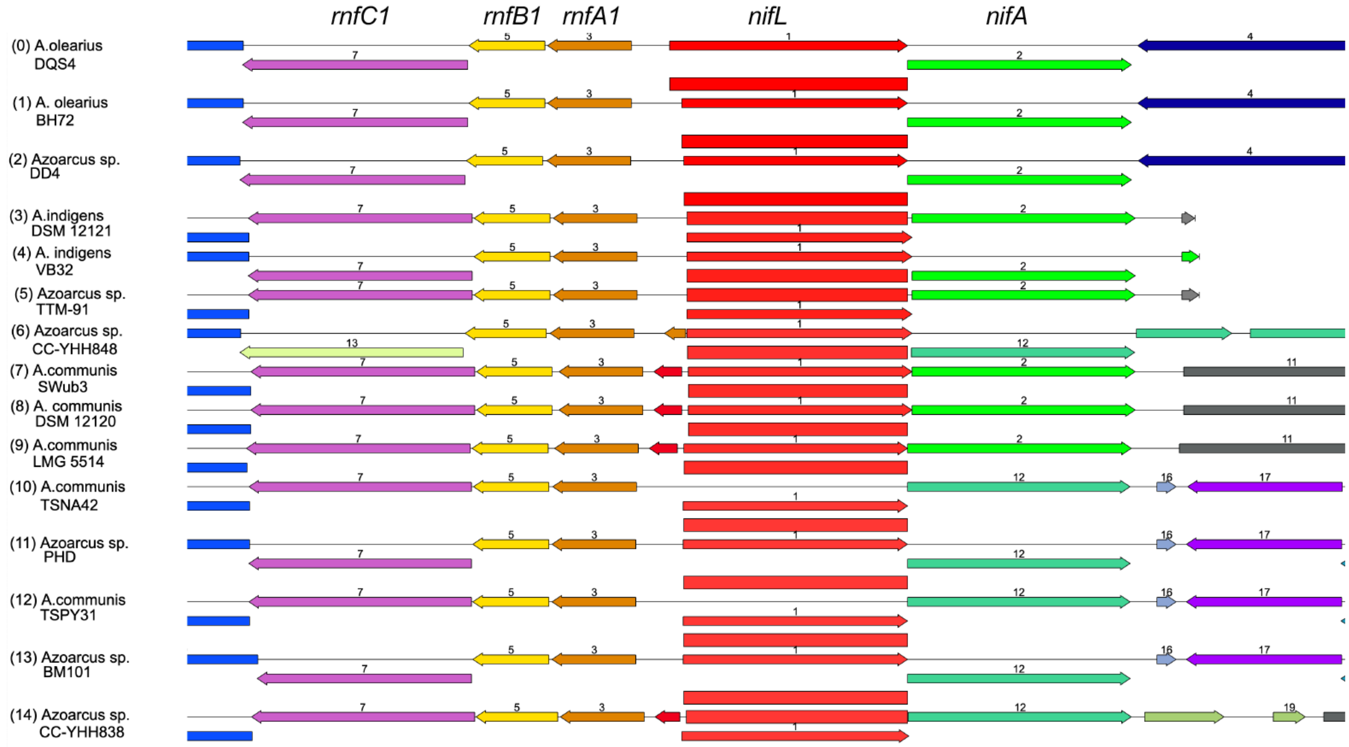
rnfC1
rnfB1
rnfA1
nifL
nifA
(0) A.olearius DQS4
(1) A. olearius BH72
(2) Azoarcus sp. DD4
(3) A.indigens DSM 12121
(4) A. indigens VB32
(5) Azoarcus sp. TTM-91
(6) Azoarcus sp. CC-YHH848
(7) A.communis SWub3
(8) A. communis DSM 12120
(9) A.communis LMG 5514
(10) A.communis TSNA42
(11) Azoarcus sp. PHD
(12) A.communis TSPY31
(13) Azoarcus sp. BM101
(14) Azoarcus sp. CC-YHH838

B.

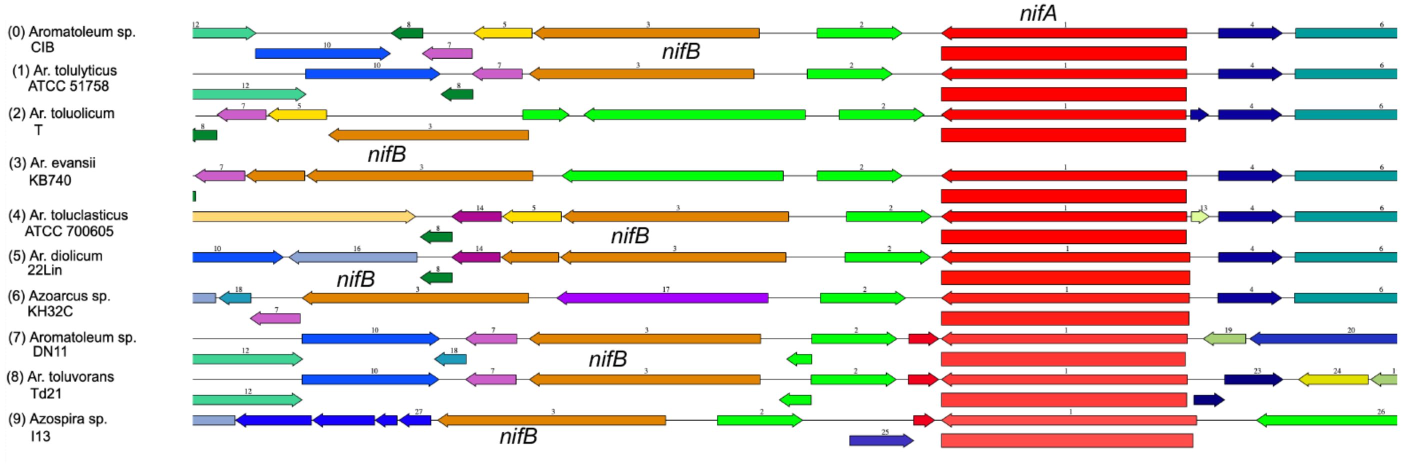
nifA
nifB
(0) Aromatoleum sp. CIB
(1) Ar. tolulyticus ATCC 51758
(2) Ar. toluolicum T
(3) Ar. evansii KB740
(4) Ar. toluclasticus ATCC 700605
(5) Ar. diolicum 22Lin
(6) Azoarcus sp. KH32C
(7) Aromatoleum sp. DN11
(8) Ar. toluvorans Td21
(9) Azospira sp. I13
